# Role of miR‐15a‐5p and miR‐199a‐3p in the inflammatory pathway regulated by NF‐κB in experimental and human atherosclerosis

**DOI:** 10.1002/ctm2.1363

**Published:** 2023-08-21

**Authors:** Paula González‐López, Marta Álvarez‐Villarreal, Rubén Ruiz‐Simón, Andrea R. López‐Pastor, Melina Vega de Ceniga, Leticia Esparza, José L. Martín‐Ventura, Óscar Escribano, Almudena Gómez‐Hernández

**Affiliations:** ^1^ Hepatic and Vascular Diseases Laboratory. Biochemistry and Molecular Biology Department, School of Pharmacy Complutense University of Madrid Madrid Spain; ^2^ Department of Angiology and Vascular Surgery Hospital of Galdakao‐Usansolo Galdakao Bizkaia Spain; ^3^ Biocruces Bizkaia Health Research Institute Barakaldo Bizkaia Spain; ^4^ IIS‐Fundation Jimenez‐Diaz Autonoma University of Madrid and CIBERCV Madrid Spain

**Keywords:** atherosclerosis, inflammation, miRNAs, NF‐κB

## Abstract

**Background:**

Cardiovascular diseases (CVDs) prevalence has significantly increased in the last decade and atherosclerosis development is the main trigger. MicroRNAs (miRNAs) are non‐coding RNAs that negatively regulate gene expression of their target and their levels are frequently altered in CVDs.

**Methods:**

By RT‐qPCR, we analysed miR‐9‐5p, miR‐15a‐5p, miR‐16‐5p and miR‐199a‐3p levels in aorta from apolipoprotein knockout (*ApoE^−/−^*) mice, an experimental model of hyperlipidemia‐induced atherosclerosis, and in human aortic and carotid atherosclerotic samples. By in silico studies, Western blot analysis and immunofluorescence studies, we detected the targets of the altered miRNAs.

**Results:**

Our results show that miR‐15a‐5p and miR‐199a‐3p are significantly decreased in carotid and aortic samples from patients and mice with atherosclerosis. In addition, we found an increased expression in targets of both miRNAs that participate in the inflammatory pathway of nuclear factor kappa B (NF‐κB), such as IKKα, IKKβ and p65. In human vein endothelial cells (HUVECs) and vascular smooth muscle cells (VSMCs), the overexpression of miR‐15a‐5p or miR‐199a‐3p decreased IKKα, IKKβ and p65 protein levels as well as NF‐κB activation. On the other hand, miR‐15a‐5p and miR‐199a‐3p overexpression reduced ox‐LDL uptake and the inflammation regulated by NF‐κB in VSMCs. Moreover, although miR‐15a‐5p and miR‐199a‐3p were significantly increased in exosomes from patients with advanced carotid atherosclerosis, only in the ROC analyses for miR‐15a‐5p, the area under the curve was 0.8951 with a *p* value of .0028.

**Conclusions:**

Our results suggest that the decrease of miR‐199a‐3p and miR‐15a‐5p in vascular samples from human and experimental atherosclerosis could be involved in the NF‐κB activation pathway, as well as in ox‐LDL uptake by VSMCs, contributing to inflammation and progression atherosclerosis. Finally, miR‐15a‐5p could be used as a novel diagnostic biomarker for advanced atherosclerosis.

## Graphical abstract

This study demonstrates a novel role for miR‐15a‐5p and miR‐199a‐3p in atherosclerosis progression. Both miRNAs have an anti‐inflammatory role due to the regulation of their targets involved NF‐kappa B pathway, in addition, they have an anti‐foaming role in vascular smooth muscle cells. Finally, miR‐15a‐5p might be useful for diagnosis of advanced carotid atherosclerosis.

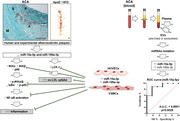

## 1 INTRODUCTION

Cardiovascular diseases (CVDs) and cancer are the dominant causes of death in the world.^1^ Atherosclerosis is the main cause of CVDs including heart failure, stroke, myocardial infarction and peripheral arterial disease.^2^ Atherosclerosis is located in the intima of many middle sized and large arteries, especially in the locations with geometrically complex vessels.^2^ Laminar and unidirectional flow induces an increase in transcription factors such as Kruppel‐like factors (KLF2 and KLF4) and nuclear factor erythroid 2‐related factor 2 (Nrf2) that will favour an atheroprotective phenotype.^3^ However, non‐uniform, turbulent, multidirectional or oscillatory flow regions, as in the bifurcation of the carotid artery produces an increase of inflammatory transcription factors and degradation of endothelial glycocalyx, leading to atherosclerotic phenotype.^3^, ^4^

Inflammation is a key factor in the progression of atherosclerosis and in the rupture of advanced atherosclerotic plaques.^5^ The dysfunctional and activated endothelium increases the secretion of chemokines, such as monocyte chemoattractant protein‐1 (MCP‐1), which favours the recruitment and diapedesis of monocytes and the different lymphocytes, as well as adhesion molecules and selectins that favour rolling, and transmigration in the subendothelial space. These monocytes in the subendothelial space will differentiate into macrophages. Mainly, there have been identified two populations of macrophages, M1 and M2, being the first one highly expressed in symptomatic patients with advanced carotid atherosclerosis (ACA).^6^ Moreover, other immune‐inflammatory cells as dendritic cells or lymphocytes T and B also participate in the development of atherosclerosis.^5^ In addition, many of the proinflammatory mediators, enzymes, chemokines and cytokines involved in atherogenesis could be regulated by different transcription factors, among them the nuclear factor kappa B (NF‐κB).^7^

NF‐κB is formed by p50 and p65, and IκB subunits in the cytosol. The phosphorylation of inhibitory subunit, IκB, by IKK complex (IKKα/IKKβ/IKKγ), its polyubiquitination, and finally, degradation by proteasome, permit the p50/p65 heterodimer nucleus translocation,^7^ which activates the transcription of genes involved in the inflammatory, immune or acute‐phase response. Increased NF‐κB activation has been found in peripheral blood mononuclear cells (PBMCs) from patients with unstable angina^8^ or acute coronary syndrome.^9^ Moreover, patients with ACA also showed a significant increase of active NF‐κB in atherosclerotic lesions and in PBMCs.^10^, ^11^

MicroRNAs (miRNAs) are small endogenous non‐coding RNAs that negatively regulate the translation of mRNAs. miRNAs play a role in development, metabolism, cell proliferation, growth, differentiation and death.^12^ However, it has been described that the alteration in the expression profile of miRNAs might be related to human diseases, such as atherosclerosis. For instance, miR‐145 and miR‐143 regulate vascular smooth muscle cells (VSMCs) fate and plasticity,^13^ whereas miR‐24‐3p has been described as an important regulator in VSMCs proliferation and apoptosis.^14^

Atherosclerosis is an asymptomatic disease for long periods in which several factors can contribute to the progression and rupture of vulnerable plaques and, consequently, provoke the acute event such as stroke or acute myocardial infarction.^15^ In this regard, to identify new biomarkers, miRNAs or miRNA panels will help in the future to detect the presence of vulnerable plaques, but also to avoid the progression process. After a screening of 20 miRNAs involved in inflammation and metabolic diseases such as NAFLD and atherosclerosis, in this manuscript, we have deepened the study of two miRNAs (miR‐15a‐5p and miR‐199a‐3p) that showed a similar behaviour in vascular samples from human and experimental atherosclerosis. We have analysed the expression of these miRNAs by RT‐qPCR in human atherosclerotic samples, using aortic samples from control subjects (CAs) and subjects with fibrolipidic plaques (FAs) or carotid samples from patients with ACA. Then, these miRNAs were also studied in the aorta from *ApoE* ^−/−^ mice (a classical experimental model of atherosclerosis) under a standard diet (STD) or a high‐fat diet (HFD) for 8 and 18 weeks and wild‐type mice. So, we focused on the role of miR‐15a‐5p and miR‐199a‐3p and their targets (IKKα, IKKβ and p65) in the progression of experimental and human atherosclerosis, as well as their implication in inflammation and NF‐κB activation. To further characterize the role of these miRNAs in atherosclerosis progression, we performed overexpression experiments in human vein endothelial cell (HUVECs) and VSMCs. Finally, we analysed the utility of circulating miR‐15a‐5p and/or miR‐199a‐3p levels as diagnostic biomarkers of human advanced atherosclerosis.

## 2 METHODS

### 2.1 Human samples

Two cohorts of patients were analysed in this study. In the first cohort, under the authorization of the French Biomedicine Agency (PFS 09‐007) human aortas were collected from deceased organ donors from 2010 to 2013. After macroscopic evaluation, the aortas were classified following the Stary classification^15^ into two groups: control aortas (CAs, *n* = 7), and aortas with initial fibrolipidic plaques (fibroatheromas [FAs], *n* = 7) as previously described.^16^ The investigation conforms to the principles outlined in the Declaration of Helsinki.

The second cohort includes patients with ACA. Patients with carotid stenosis >70% underwent carotid endarterectomy at IIS‐Fundación Jiménez Díaz (Table S1) and the atherosclerotic plaques (*n* = 40) were collected for further analysis. The plaques showed higher inflammatory cells infiltration (Stary stages V–VI), however, the adjacent areas showed mainly lipid depots and VSMCs (Stary stage III). In the same study, plasma was collected from 29 patients to obtain extracellular vesicles and analyse miRNAs levels (Table S1). The study was approved by the Hospital's Ethics Committee (IIS‐Fundación Jiménez Díaz) with reference number PI1442016 according to the institutional and the Good Clinical Practice guidelines, which was performed in accordance with the Declaration of Helsinki. All participants gave written informed consent.

### 2.2 Animal model

Male C57Bl/6 wild type (WT) and ApoE deficient (*ApoE* ^−/−^) mice were maintained under standard light (12 h long light/dark cycles), temperature (23.3°C) and humidity (65.1%) conditions, and ad libitum diet from their weaning, up to their sacrifice. The WT mice (*n* = 7) were fed a standard type diet (STD; 3% of the kcal are provided by fat, Envigo, USA) for 8 or 18 weeks, while the *ApoE* ^−/−^ mice were separated into two groups: one was fed the STD (*n* = 7) and the other a HFD (*n* = 10) for 8 or 18 weeks before sacrifice. The formula of HFD (TD06414, Envigo, USA) is composed of casein (265.0 g/kg), l‐cystine (4.0 g/kg), maltodextrin (60.0 g/kg), sucrose (90.0 g/kg), lard (310.0 g/kg), soybean oil (30.0 g/kg), cellulose (65.5 g/kg), mineral mix (AIN‐93G‐MX (94046), 48.0 g/kg), calcium phosphate dibasic (3.4 g/kg), vitamin mix (AIN‐93‐VX (94047), 21.0 g/kg) and choline bitartrate (3.0 g/kg). The percentage of kcal supported by protein (18.3%), carbohydrate (27.3%) and fat (60.3%). Fatty acid profile (percentage of total fat): 36% saturated, 41% monounsaturated and 23% polyunsaturated.

Mice were sacrificed at 8 or 18 weeks of feeding with STD or HFD, after 16 h of fasting. All animal experimentations were conducted in accordance with Complutense University of Madrid Ethics Committee, Autonomic Community of Madrid (PROEX133/19) and the guidelines from Directive 2010/63/EU of the European Parliament regarding the protection of animals used for scientific purposes. The microbiological and health state of the mice was controlled by the Federation of European Laboratory Animal Science Associations (FELASA) criteria and showed no pathogenic infection.

For euthanasia, the mice were anesthetized with ketamine (50 ng/mL, Ketalar^®^ [Pfizer, New York, USA]) and xylazine (200 mg/mL Rompun^®^ [Bayer, Barmen, Germany]) intraperitoneal injection on a 50:5 dose per Kg. The aorta was harvested and stored at −80°C, while the aortic root was washed with saline and then included in Tissue‐Tek^®^ O.C.T. Compound ([O.C.T.], VWR BDH Chemicals^®^, Radnor, PA, USA) and stored at −80°C for further analysis. Both tissues were extracted under sterile conditions. Blood was extracted from the jugular vein and mixed with 0.4% p/v citrate (Merck, Darmstadt, Germany), then the plasma was recovered after a 1200 × *g* centrifuge for 15 min at 4°C for subsequent analysis. Before the injection, the animals were weighted, and plasma glucose was measured using an Accu‐Chek^®^ glucometer (Aviva Roche, Basel, Switzerland). Finally, cholesterol and triglycerides were tested in plasma samples from fasted mice (Spinreact, Girona, Spain).

### 2.3 Cell culture

HUVECs were purchased from PromoCell. They were grown in MCDB‐131 culture medium (Life Technologies, Carlsbad, CA, USA) enriched with l‐glutamine 2 mM (GibcoTM, Fisher Scientific, Hampton, NH, USA), foetal bovine serum (FBS) 7.5% v/v (GibcoTM, Fisher Scientific, Hampton, NH, USA), Penicillin/Streptomycin 100 U/mL (GibcoTM, Fisher Scientific, Hampton, NH, USA) and endothelial growth factor 1X (R&D systems^®^, Minneapolis, MN, USA). Cells were received at passage 2 and were grown until passage 5. All cells were grown at 37°C in a humidified 5% CO_2_ incubator (Fisher Scientific, Hampton, NH, USA).

Generation of immortalized WT VSMCs lines was previously described.^17^ Cell lines were cultured to subconfluence (70%−80%) with 10% FBS‐DMEM for in vitro experiments.

### 2.4 Histological tissue samples

Paraffin‐embedded human carotids were cut into 5 μm sections and stained with Masson trichrome and contrasted with haematoxylin and eosin purchased from PanReac Appli‐Chem ITW Reagents (PanReac Appli‐Chem ITW Reagents, Darmstadt, Germany). IKKα, IKKβ, p65 and LOX‐1 were detected by immunoperoxidase with rabbit anti‐IKKα (sc‐7606, Santa Cruz Biotechnology, Dallas, TX, USA), anti‐IKKβ (#2678, Cell Signalling Technology Inc.^®^, Danvers, MA, USA), anti‐LOX‐1 (#PA5‐102452, Invitrogen, Waltham, MA, USA) and anti‐p65 (#PA1‐186, Invitrogen, Waltham, MA, USA) polyclonal antibodies (see Table S2). After an overnight incubation with each primary antibody, sections were incubated with a peroxidase‐conjugated secondary antibody for 1 h at 1:100 dilution or biotin‐conjugated secondary antibody (1:200) (Table S2). When we used peroxidase‐conjugated secondary antibodies, the slides were incubated with peroxidase substrate DAB (416424, Palex, Barcelona, Spain) for 30 min. For the IKKα and LOX‐1, an incubation of 30 min with Vectastain Elite ABC‐HRP Kit (416411, Palex, Barcelona, Spain) at room temperature was carried out after the secondary antibody incubation and before the DAB incubation. The sections were stained for 10 min with DAB at room temperature and then counterstained with haematoxylin and mounted in DPX mounting medium (255,254.1610, PanReac Appli‐Chem ITW Reagents, Darmstadt, Germany). In each experiment, we included negative controls without the primary antibody to check for non‐specific staining.

The immunohistochemistry images were quantified using the “count and measure objects” tool in the Image‐Pro Plus software IPWin (v4.5, Media Cybernetics, Rockville, USA) (IPWin v4.5 software). The colour considered as positive staining for the same protein was manually selected and all samples quantified with the same parameters, and the value corresponding to the sum of all stained areas was obtained. The results were expressed as the percentage of the stained area with respect to the total area analysed in each sample.

### 2.5 En face imaging of aorta

We quantified the atherosclerotic lesions of the whole aorta were quantified by en face analysis. For *it*, the aorta was opened longitudinally, while still attached to the heart and major branching arteries in the body. The aorta from the heart to the iliac bifurcation was then removed and was pinned out on a white wax surface in a dissecting pan using stainless steel pins 0.2 mm in diameter. After overnight fixation with 4% paraformaldehyde and PBS rinsing, the aortas were stained for 6 min in a filtered solution containing 0.5% Oil Red O, 35% ethanol and 50% acetone, and then destained in 80% ethanol. The Oil Red O stained aortas were photographed, and the atherosclerotic lesions were quantified using IP Win32 v4.5 software.

### 2.6 miRNA extraction from the aorta, vascular cell lines and paraffin‐embedded carotid tissue

The miRNA content from the aorta and the cells were extracted following the mirVanaTM miRNA Isolation Kit (InvitrogenTM, Thermo Fisher Scientific, Waltham, MA, USA). The miRNA content from paraffin‐embedded carotids was extracted using the RNeasy FFPE kit (Qiagen, Hilden, Germany). All the extractions were made following the protocol handled by the manufacturers. The mirVana^™^ kit allows the isolation of miRNAs and long RNAs in separate fractions by differential precipitation. The miRNA sample concentration was determined using a NanoDropTM 2000 and the NanoDrop 2000/2000c Operating Software (Thermo Scientific, Waltham, MA, USA).

### 2.7 Cell transfection with miRNA precursors

Precursors of miR‐15a‐5p and miR‐199a‐3p were purchased from Sigma‐Aldrich. Approximately 5 × 10^4^ cells were seeded in P60 culture plates (353002, FalconTM, Thermo Fisher Scientific, Waltham, MA, USA) and transfected with 10–20 nM of MISSION^®^ miRNA mimic hsa‐miR15a‐5p or 50 nM hsa‐miR‐199a‐3p (HMI0256 or HMI0340, Sigma‐Aldrich, St. Louis, MO, USA). As specified in the manufacturer's protocol (#409‐10, Polyplus transfection^®^, Strasbourg, France), miRNA expression in HUVECs was assessed 72 h after transfection, whereas protein downregulation was analysed 96 h following transfection. In VSMCs, the effect of the transfection in both miRNA and protein levels was assessed after 48 h transfection with Lipofectamine^®^2000 RNAiMAX. To evaluate the effect in NF‐κB pathway in cells transfected with pre‐miR‐15a‐5p or/and miR‐199a‐3p, they were deprived in 0% FBS medium for 1 h and then stimulated with 10 ng/mL TNF‐α (Sigma‐Aldrich, St. Louis, MO, USA) for 10 min. To study the effect of the pre‐miR‐15a‐5p and miR‐199a‐3p on inflammation, VSMCs were treated with low density lipoprotein from human plasma, oxidized (ox‐LDL) (L34357, Thermo Fisher Scientific, Waltham, MA, USA) and some of them were transfected with the pre‐miRNAs and analysed whether there was a less LDL uptake, reduced NF‐κB activation as well as gene target regulated by above nuclear transcription factor.

On the other hand, we have also used hsa‐antagomiR15a‐5p (MSTUD0211, Sigma‐Aldrich, St. Louis, MO, USA) and hsa‐antagomiR‐199a‐3p (MSTUD0113, Sigma‐Aldrich, St. Louis, MO, USA) to demonstrate the effect of the decrease of miR‐15a‐5p or miR‐199a‐3p levels on NF‐κB activation. The effects of the transfection in both, miRNAs and protein levels, were assessed after 48 h of transfection with Lipofectamine^®^2000 RNAiMAX. To evaluate the effect in NF‐κB pathway in cells transfected with hsa‐antagomiR‐15a‐5p (20 nM) or hsa‐antagomiR‐199a‐3p (20 nM), they were deprived in 0% FBS medium for 1 h and then stimulated with 10 ng/mL TNF‐α (Sigma‐Aldrich, St. Louis, MO, USA) for 10 min.

### 2.8 Oil Red O staining of vascular smooth muscle cells

After the 100 μg/mL ox‐LDL treatment in the last 24 h of transfection with the pre‐miR‐15a‐5p and pre‐miR‐199a‐3p, the cells were carefully washed two times with PBS 1X and put on ice. To fix the cells, they were incubated for 20 min with 10% formalin. The formalin was removed with PBS 1X. The cells were quickly washed with 65% isopropanol before the 15‐min incubation with Oil Red O stock solution (0.5 g of Oil Red in 100 mL of 99% of 2‐propanol, then we added 40 mL of water). We wait for 10−15 min before filtering and then washed with 65% isopropanol again. Finally, the cells were washed with distilled water to remove the excess colouring. The pictures were taken with a Nikon TE300 Inverted Phase Contrast DIC Fluorescence Microscope using a Nikon digital sight camera and the Nikon NIS‐Elements F software (v 5.22.00 64‐bit). The LDL uptake was quantified using ImageJ‐win 64 software. All the pictures were open at the same time and the image type was changed to 8‐bit, then the same threshold was asset for all the pictures to detect lipid deposition, and the selected staining from the images was measured at the same time.

### 2.9 RT‐qPCR analysis

Complementary DNA (cDNA) was synthesized by a High‐Capacity cDNA Reverse Transcription Kit (Applied Biosystems, Foster City, CA, USA) for mRNA analysis. Quantitative polymerase chain reaction (qPCR) was done using cDNA as template and the TaqMan^®^ Fast Advanced Master Mix (Thermo Scientific, Waltham, MA, USA). The genes were detected using TaqMan^®^ (Thermo Scientific, Waltham, MA, USA) probes for mmu‐miR‐15a‐5p (mmu482962_mir, mature miRNA sequence: UAGCAGCACAUAAUGGUUUGUG), hsa‐miR‐199‐3p (477961_mir, mature miRNA sequence: ACAGUAGUCUGCACAUUGGUUA), mmu‐miR‐9a‐5p (mmu481285_mir, mature miRNA sequence: UCUUUGGUUAUCUAGCUGUAUGA) and mmu‐miR‐16‐5p (mmu482960_mir, mature miRNA sequence: UAGCAGCACGUAAAUAUUGGCG) was used as an endogenous gene in plasma samples, and mmu‐miR‐191‐5p (mmu481584_mir, mature miRNA sequence: CAACGGAAUCCCAAAAGCAGCUG) used as endogenous gene in the other analysis. All the probes detect both mouse and human target genes. All RT‐qPCR experiments were performed in an ABI Prism 7900HT Thermal Cycler (Applied Biosystems, Foster City, CA, USA).

The relative abundance of mRNA targets, normalized with the endogenous gene and relative to the control, is calculated as follows: Relative quantification (RQ) = 2−ΔΔCt; ΔCt (cycle threshold) = Ct (miRNA target) − Ct (miR‐191‐5p); ΔΔCt = [ΔCt (for any sample) − ΔCt (for the control)]. Amplification of miR‐191‐5p was used in the same reaction of all samples as an internal control.

### 2.10 Western blot analysis

Proteins from cell lysates (20–40 μg), and tissue samples (60 μg) were separated on a 10% or 10%−20% gradient acrylamide gel and then transferred to a 0.45 μM pore PVDF membrane (Merck, Darmstadt, Germany) as previously described.^16^, ^17^ The primary antibodies used are shown in Table S2 and all of them were diluted in TTBS. Rabbit and mouse primary antibodies were immunodetected using horseradish peroxidase‐conjugated anti‐rabbit IgG (NA931V; 1:4000 in TTBS) or anti‐mouse IgG secondary antibody (NA934V; 1:5000 in TTBS) (GE Healthcare, Buckinghamshire, UK), respectively. When possible, phosphoproteins and their total expression were detected in the same gel, using Restore^™^ Western Blot Stripping Buffer (Thermo Fisher Scientific) as per the manufacturer's instructions, blocking the membrane again before the incubation with the next antibody. Loading was normalized by β‐actin or α‐tubulin. Protein bands were visualized using the SuperSignal^™^ West Pico PLUS Chemiluminescent Substrate (34580, Thermo Fisher Scientific^®^, Hercules, CA, USA). Band densitometry was analysed using ImageJ Software (v1.52a, Wayne Rasband, National Institute of Health, Stapleton, USA).

### 2.11 Immunofluorescence

The coverslips were pretreated with 0.2% gelatin for 30 min at room temperature and rinsed twice with PBS 1X in a 24‐well culture plate, then 15 × 10^3^ HUVECs were seeded for transfection. After the 96 h transfection with pre‐miR‐15a‐5p or/and pre‐miR‐199a‐3p followed by a 1 h deprivation and the stimulation with 10 ng/mL TNF‐α, the cells were rinsed twice with PBS 1X and fixed with 4% paraformaldehyde (252931.1214, PanReac AppliChem, ITW Reagents, Glenview, IL, USA) for 20 min, then the cells were rinsed twice with PBS 1X again and permeabilized with Triton X‐100 0.5% and SDS 0.1% for 5 min each. Afterwards the cells were blocked with PBS 1X—4% BSA (A6588,0100, PanReac AppliChem, ITW Reagents, Glenview, IL, USA)—1.5% normal goat serum (1000 C, Invitrogen, Waltham, MA, USA) for 30 min at room temperature, then the p65 NF‐κB primary antibody was diluted at 1:200 of the blocking buffer and was incubated at 4°C overnight. After the primary antibody incubation, the cells were rinsed three times with PBS 1X, and were then incubated with the secondary antibody 555 goat anti‐rabbit (A32732, Invitrogen, Waltham, MA, USA) diluted at 1:500 and DAPI (A4099, Sigma‐Aldrich, Darmstadt, Germany) at 1:1000 of the blocking buffer, for 1 h at room temperature. Finally, the cells were rinsed twice with PBS 1X and once with distilled water and the coverslips were mounted with ProLong^™^ Gold antifade reagent mounting medium (P36930, Invitrogen, Waltham, MA, USA) and inverted onto glass slides. The pictures were taken with a Nikon TE300 Inverted Phase Contrast DIC Fluorescence Microscope using a Nikon HB‐10101AF super high pressure mercury lamp power supply and a Nikon digital sight camera and the Nikon NIS‐elements F software (v 5.22.00 64‐bit) and Fluorescence microscopy (Leica SP‐2 AOBS). The co‐localization of p65/DAPI and DAPI/p65 was calculated with the JaCoP plugin from the Image J‐win64 Software to calculate the M1, M2 and Pearson's coefficients.

### 2.12 Nuclear fractionation and extraction of proteins

HUVECs were maintained in MCDB‐131 medium with 0.5% FBS for at least 18 h, then stimulated with TNF‐α (10 ng/mL) for 10–40 min. Lysates of endothelial cells were resuspended in a buffer, which consisted of 10 mM HEPES (pH 7.8), 15 mM KCl, 2 mM MgCl_2_, 0.1 mM EDTA, 1 mM dithiothreitol (DTT) and 1 mM phenylmethylsulfonyl fluoride. After 10 min on ice, the lysates were pelleted and resuspended in 2 volume of the buffer. Then, 3 M KCl was added dropwise to reach a 0.39 M KCl concentration. We extracted the nuclei from the cells with incubation for 1 h at 4°C followed by centrifugation at 12,000 × *g* for 1 h. The supernatants were then dialyzed in a buffer containing 50 mM HEPES (pH 7.8), 50 mM KCl, 0.1 mM EDTA, 1 mM DTT and 1 mM phenylmethylsulfonyl fluoride with 10% (v/v) glycerol. The samples were then cleared by centrifugation and stored at −80°C until further use. Total protein concentration was determined by the Bradford method (Thermo Fisher Scientific). We analysed the levels and phosphorylation of IκBα, IKKα and IKKβ in cytosolic fractions by Western blotting as described above. We measured p65 in the nuclear and cytosol fractions by Western blot studies. We used β‐actin and histone H3 as control for total protein in cytosolic and nuclear fractions, respectively.

### 2.13 Luciferase reporter assays

For reporter assays, a region of wild‐type 3′‐untranslated region (3′UTR) from *IKBKB* (miR‐15a‐5p), the 3′UTR from mutated *IKBKB* (miR‐15a‐5p), the wild‐type 3′UTR from *IKBKB* (miR‐199a‐3p), the 3′UTR from mutated *IKBKB* (miR‐199a‐3p), the wild‐type 3′UTR from *CHUK* (miR‐15a‐5p), the 3′UTR from mutated *CHUK* (miR‐15a‐5p), the wild‐type 3′UTR from *RELA* (miR‐199a‐3p) and the 3′UTR from mutated *RELA* (miR‐199a‐3p) were constructed annealing the following primers:

3′UTR‐*IKBKB* (miR‐15a‐5p) F: 5′‐TCGAGACTGACCTCTTTTTATTTCACTGCTG CTATATTAAAAGGAGTATGC‐3′ and 3′UTR‐*IKBKB* (miR‐15a‐5p) R: 5′‐GGCCGCA TACTCCTTTTAATATAGCAGCAGTGAAATAA AAAGAGGTCAGTC‐3′; 3′UTR‐*IKBKB* (miR‐15a‐5p) mutated. F: 5′‐TCGAGACTGACCTCTTTTTATTTCACCCCTTTGCTATTAAA AGGAGTATGC‐3′ and 3′UTR‐*IKBKB* (miR‐15a‐5p) mutated. R: 5′‐GGCCGC ATACTCCTTTTAATAGCAAAGGGGTGAAATAAAAAGAGGTCAGTC‐3′.

3′UTR‐*IKBKB* (miR‐199a‐3p) F: 5′‐TCGAGACTGACCGCCTTGTCTGCACACTGGAGG TCCTCCATTTAT TAAAAGGAGTATGC‐3′ and 3′UTR‐*IKBKB* (miR‐199a‐3p) R: 5′‐GGCCGCATACTCCTTTTAATAAATGGAGGACCTCCAGTGTGCAGACAAGGCGGTCAGTC‐3′; and 3′UTR‐*IKBKB* (miR‐199a‐3p) mutated F: 5′‐TCGAGACTGACCGGC GCCTTGTCTGCGAGGACCATATTAAAAGGAGTATGC‐3′ and 3′UTR‐*IKBKB* (miR‐199a‐3p) mutated R: 5′‐GGCCGCATACTCCTTTTAATATGGTCCTCGCAGACAAGGCGCC GGTCAGTC‐3′.

3′UTR‐*CHUK* (miR‐15a‐5p) F: 5′‐TCGAGACTGACGAATATTCCTTTATTTTGCTGCT TATTAAAAGGAGTATGC‐3′ and 3′UTR‐*CHUK* (miR‐15a‐5p) R: 5′‐GGCCGCATA CTCCTTTTAATAAGCAGCAAAATAAAGGAATATTCGTCAGTC‐3′; and 3′UTR‐*CHUK* (miR‐15a‐5p) mutated F: 5′‐TCGAGACTGACGAATATTCCTTTATTTCTGTAAGTAT TAAAAGGAGTATGC‐3′ and 3′UTR‐*CHUK* (miR‐15a‐5p) mutated R: 5′‐GGCCGCATACT CCTTTTAATACTTACAGAAATAAAGGAATATTCGTCAGTC‐3′.

3′UTR‐*RELA* (miR‐199a‐3p) F: 5′‐TCGAGACTGACAGGTGACAGTGCGGGACC CATCAGTATTAAAAGGAGTATGC‐3′ and 3′UTR‐*RELA* (miR‐199a‐3p) R: 5′‐GGCCGCATACTCCTTTTAATACTGATGGGTCCCGC ACTGTCACCTGTCAGTC‐3′; and 3′UTR‐*RELA* (miR‐199a‐3p) mutated F: 5′‐TCGAGACTGACAGGTGACAGTGCGG GACCAGGCCATATTAAAAGGAGTATGC‐3′ and 3′UTR‐*RELA* (miR‐199a‐3p) mutated R: 5′‐GGCCGCATACTCCTTTTAATATGGCCTGGTCCCGCACTGTCACCTGTCAGTC‐3′.

Annealing was conducted by incubating both primers for 4 min at 95°C and for 10 min at 70°C in annealing buffer (100 mM potassium acetate, 30 mM HEPES, pH 7.4 and 2 mM magnesium acetate). Primers were then phosphorylated and cloned into the psiCHECK2 vector from Promega digested with *Xho*I and *Not*I.

Thirty thousand HEK293 cells were plated in DMEM containing 10% FBS. Twenty‐four hours later, cells were transfected with the psiCHECK2 vectors either with pre‐miR control, pre‐miR‐15a‐5p or pre‐miR‐199a‐5p using lipofectamine (Invitrogen) following the manufacturer's instructions. The luciferase reporter assay was performed 72 h after transfection using the Dual‐Glo Luciferase Kit (Promega, Madison, WI, USA).

The ratio between the firefly and the *Renilla* luciferase allows the normalization of luciferase values. Ratios were normalized against the ratio of the corresponding plasmid transfected with the miR‐Control. Statistical differences were determined using unpaired *t*‐test.

### 2.14 Extraction of exosomes of plasma from patients with ACA and controls

To precipitate the exosomes from the plasma samples the total exosome RNA and protein isolation kit (from plasma) (4478545, Thermo Fisher Scientific, Waltham, MA, USA), were used following the protocol recommended by the manufacturer. First, the plasma was clarified with one centrifugation at 2000 × *g* for 20 min, and a second centrifugation at 10,000 × *g* for 20 min, in both cases the supernatant was collected, and the pellet was discharged. When the plasma was ready, we added 0.5 volume of PBS 1X and 0.2 volume of the Exosome Precipitation Reagent (from plasma) (4484451, Thermo Fisher Scientific, Waltham, MA, USA) mixing until the sample becomes cloudy, at this point the mix was incubated for 30 min on ice followed by a centrifugation at 10,000 × *g* for 30 min at room temperature. The pellet containing the exosomes was used to extract the miRNAs following the mirVanaTM miRNA Isolation Kit. To confirm the pellet was enriched in exosomes a diffuse light scattering analysis and Western blot analysis of CD81, CD63 and GM130 markers were performed.

### 2.15 Database search to find miRNAs and their possible targets

The miRNAs analysed in the study were identified by an extensive search in PubMed by using the terms (miRNAs, atherosclerosis and inflammation, NF‐κB) and GEO Database. Once the miRNAs of interest were selected, the possible mRNA targets were evaluated in predictive miRNA‐target interaction databases such as, TargetScan, miRWalk, miRDB and the experimentally validated miRNA‐target interaction database: miRTarBase. The data collected from these databases were analysed, and only those targets that appeared in two or more of the predictive databases or just in miTarBase were considered as possible targets. A diagram showing the proposed regulatory axes during atherosclerosis progression is supplied in Figure S5. These representations were made using Cytoscape 3.8.2. software (v.3.8.2. on Java 11.0.6. by AdoptOpenJDK, Darmstadt, Germany) and the online tool http://www.interactivenn.net/ was used to generate the Venn diagram with the target results of each database.

### 2.16 Statistical analysis

The data from the experimental groups were analysed using the GraphPad Prism v8.2.1 software (GraphPad Software, San Diego, CA, USA). Normality and lognormality tests were performed to confirm that the data followed a normal distribution. Statistical significance of the differences between groups was assessed by Student's *t*‐tests when comparing two groups, or with ANOVA tests followed by a Bonferroni post hoc test when comparing more than two groups. Correlation between variables was assessed by two‐tailed Spearman's *r* correlation analyses. The exact *p* value is indicated in each figure when it reached statistically significance (*p* < .05).

Receiver operating characteristic (ROC) analyses were performed to test the diagnostic accuracy of the evaluated miRNAs. All statistical procedures were performed using the GraphPad Prism v8.2.1 software (GraphPad Software, San Diego, CA, USA).

## 3 RESULTS

### 3.1 miR‐15a‐5p and miR‐199a‐3p levels are decreased in human atherosclerotic carotid plaque

We aimed to identify miRNAs involved in human atherosclerosis progression. For that purpose, we collected vascular samples from control subjects (CAs), subjects with fibrolipidic plaque (FAs) and patients with human advanced atherosclerosis undergoing carotid endarterectomy (ACA) (Table S1). First, we performed Masson trichrome staining, and we could distinguish the media region in aortas from CAs and FAs and media, fibrous and shoulders in carotid from ACA ( Figure S1). The histological analysis showed that complicated plaques from ACA contained an intraplaque haemorrhage with a higher percentage of inflammatory cells and/or a certain degree of calcification. The adjacent non‐complicated regions showed a variable content of VSMCs and fibrous thickening (Figure S1).

**FIGURE 1:**
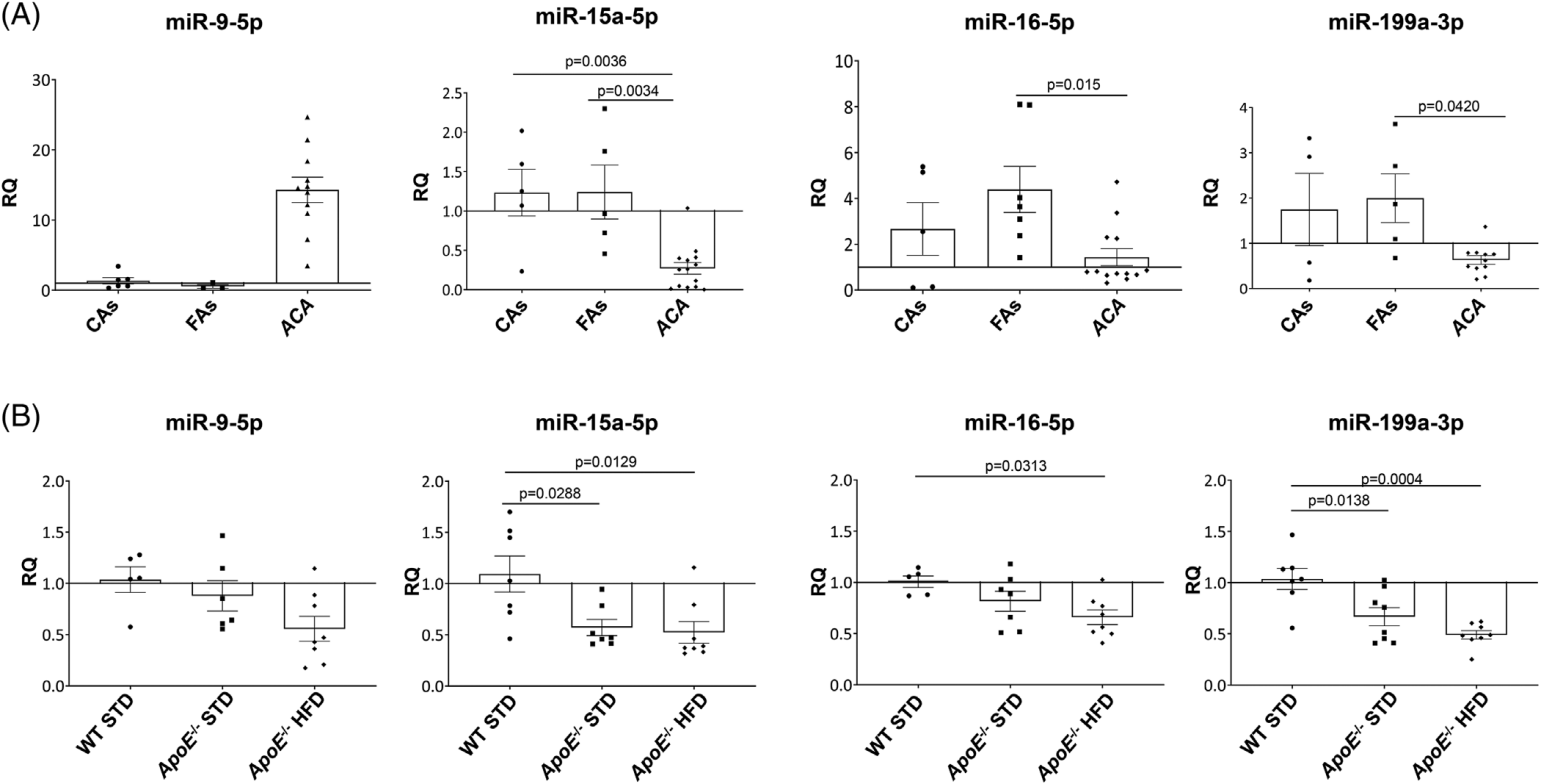
The expression of miR‐9‐5p, miR‐15a‐5p, miR‐16‐5p and miR‐199a‐3p is altered in human and experimental atherosclerosis. Relative expression of miR‐9‐5p (left), miR‐15a‐5p (middle left), miR‐16‐5p (middle right) and miR‐199a‐3p (right) in vascular human samples (A) and in the aorta of the experimental groups submitted to 18 weeks of diet (B) was measured by qPCR. Amplification of miR‐191‐5p was used in the same reaction of all samples as an internal control. ACA, advanced carotid atherosclerotic plaque patients; *ApoE* ^−/−^, *ApoE* deficient mice; CAs, control subjects; FAs, fibrolipidic plaque subjects; HFD, high‐fat diet; STD, standard type diet; WT, wild‐type group. qPCR miR‐9‐5p: WT STD (*n* = 5); *ApoE* ^−/−^ STD (*n* = 6); *ApoE* ^−/−^ HFD (*n* = 8); CAs (*n* = 6); FAs (*n* = 3); ACA (*n* = 11). qPCR miR‐15a‐5p: WT STD (*n* = 7); *ApoE* ^−/−^ STD (*n* = 7); *ApoE* ^−/−^ HFD (*n* = 8); CAs (*n* = 5); FAs (*n* = 5); ACA (*n* = 14). qPCR miR‐16‐5p: WT STD (*n* = 5); *ApoE* ^−/−^ STD (*n* = 7); *ApoE* ^−/−^ HFD (*n* = 8); CAs (*n* = 5); FAs (*n* = 7); ACA (*n* = 13). qPCR miR‐199a‐3p: WT STD (*n* = 7); *ApoE* ^−/−^ STD (*n* = 8); *ApoE* ^−/−^ HFD 18 weeks (*n* = 8); CAs (*n* = 4); FAs (*n* = 5); ACA (*n* = 11).

PubMed and GEO Databases were used to perform a search of miRNAs that could play a role in the inflammation during atherosclerosis progression. We selected and analysed 20 miRNAs involved in inflammation and metabolic diseases such as NAFLD and atherosclerosis, from that screening we selected and further study four miRNAs, miR‐9‐5p, miR‐15a‐5p, miR‐16‐5p and miR‐199a‐3p that were involved in NF‐κB pathway. Finally, we deepened in the study of miR‐15a‐5p and miR‐199a‐3p because both showed a similar behaviour in vascular samples from human and experimental atherosclerosis. In serial sections of samples used for histological characterization, we isolated and analysed miRNAs levels showing that miR‐15a‐5p expression was significantly downregulated in ACA patients compared with the other two groups, miR‐16‐5p and miR‐199a‐3p expressions were significantly decreased in ACA patients in relation to FAs subjects (Figure 1A). In contrast, miR‐9‐5p expression was upregulated in ACA patients compared with Controls and FAs, but without reaching statistical significance.

### 3.2 miR‐15a‐5p and miR‐199a‐3p levels are downregulated in aorta from *ApoE* ^−/−^ mice

We further analysed the levels of the four miRNAs studied in human atherosclerosis in the hyperlipidemic model of atherosclerosis of *ApoE^−/−^* mice under HFD. The first step was to confirm that *ApoE^−/−^* mice gained body weight, showed increased visceral and subcutaneous adiposity in addition to hypertriglyceridemia and hypercholesterolemia (Figure S2A–C) in comparison with Control STD, being significantly higher in *ApoE^−/−^* mice fed with HFD for 18 weeks. Moreover, we observed a significant increase in lesion area in *ApoE^−/−^* mice under HFD for 18 weeks versus Control STD and *ApoE^−/−^* STD of the same age by en face analysis of ORO‐stained whole aorta (Figure S2D).

A screening of four miRNAs was performed in the aorta of all the experimental groups (Figure 1B and Figure S3). We observed that miR‐15a‐5p and miR‐199a‐3p levels were significantly decreased in *ApoE^−/−^* mice after 18 weeks of HFD or STD compared with WT STD mice (Figure 1B); showing a higher decrease in aorta from *ApoE^−/−^* HFD 18 wks. Moreover, a significant decrease of miR‐16‐5p was also noted in *ApoE^−/−^* HFD in relation to WT STD (Figure 1B). However, we did not observe any significant change in miR‐9‐5p levels at 18 weeks (Figure 1B) as well as in those miRNAs studied in aorta of the three groups at 8 weeks of diet (Figure S3).

In addition, we established correlations between atherosclerosis progression (percentage lipid accumulation/aorta area or percentage lesion area/total area) and miR‐15a‐5p or miR‐199‐3p levels. We observed a significant and negative correlation between miR‐15a‐5p or miR‐199a‐3p levels and percentage lesion area or percentage lipid accumulation (Figures S4A, S4B, S4C and S4D, respectively).

Since miR‐15a‐3p and miR‐199a‐3p were altered in the same way in human and murine atherosclerosis, we focused on the study of both miRNAs and their targets in vivo and in vitro (Figure S5).

### 3.3 IKKα, IKKβ and p65 expression is modulated by both miRNAs

First, in PubMed and GEO Databases we studied the possible targets of both miRNAs regarding the inflammation, concisely, proteins involved in NF‐κB pathway (Figure S5C,D). We found as possible candidates: IKKα,^18^ IKKβ^18^ and p65.^18^, ^19^, ^20^

**FIGURE 2:**
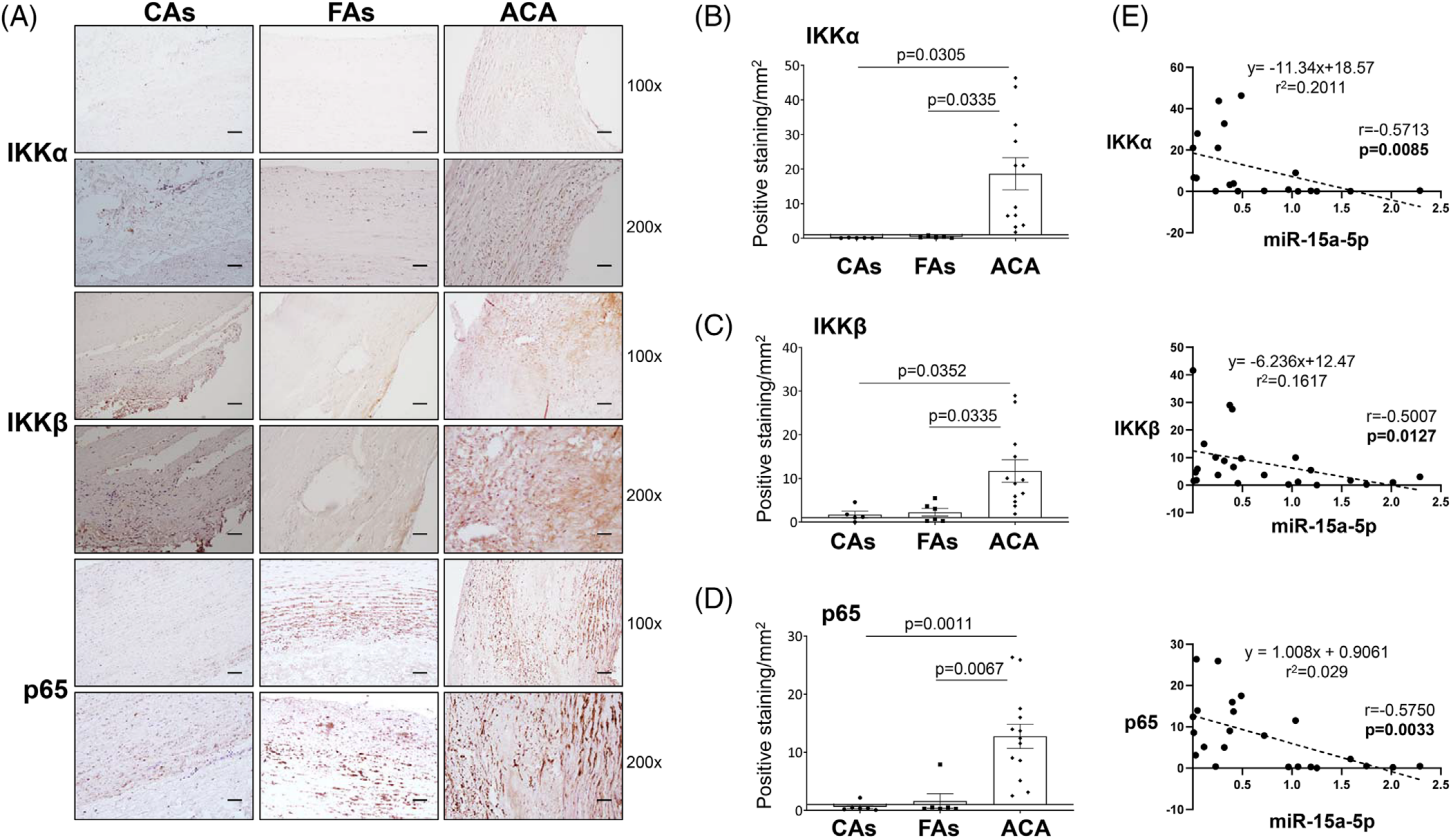
The expression of IKKα, IKKβ and p65 is upregulated in human aorta. (A) Representative images from the immunohistochemistry of IKKα (upper panels), IKKβ (middle panels) and p65 (lower panels). Magnification 100× (scale bar = 100 μm); magnification 200× (scale bar = 50 μm). The expression of IKKα (B, left), IKKβ (C, left) and p65 (D, left) was measured and the relation with miR‐15a‐5p expression (B, C and D, right) was tested by a Spearman's correlation. ACA, advanced carotid atherosclerotic plaque patients; CAs, control subjects; FAs, fibrolipidic plaque subjects; IKKα, inhibitor of nuclear factor kappa‐B kinase subunit alpha; IKKβ, inhibitor of nuclear factor kappa‐B kinase subunit beta; p65, transcription factor p65. Measurement of IKKα: CAs (*n* = 5); FAs (*n* = 5); ACA (*n* = 12). Measurement of IKKβ: CAs (*n* = 4); FAs (*n* = 6); ACA (*n* = 12). Measurement of p65: CAs (*n* = 6); FAs (*n* = 6); ACA (*n* = 13). Correlation between IKKα and miR‐15a‐5p (*n* = 20), between IKKβ and miR‐15a‐5p (*n* = 24) and between p65 and miR‐15a‐5p (*n* = 23).

Second, we checked whether their targets expression was altered in human and experimental atherosclerosis. Then, by immunohistochemistry we analysed the protein levels of IKKβ, IKKα and p65 in human vascular samples (Figure 2) and confirmed a significant increase of IKKα, IKKβ and p65 in vascular samples from ACA in relation to CAs or subjects with early atherosclerosis (Figures 2A, 2B, 2C and 2D, respectively). Moreover, we established a significant and inverse correlation between miR‐15a‐5p and its three targets (IKKα, IKKβ and p65) (Figure 2E).

**FIGURE 3:**
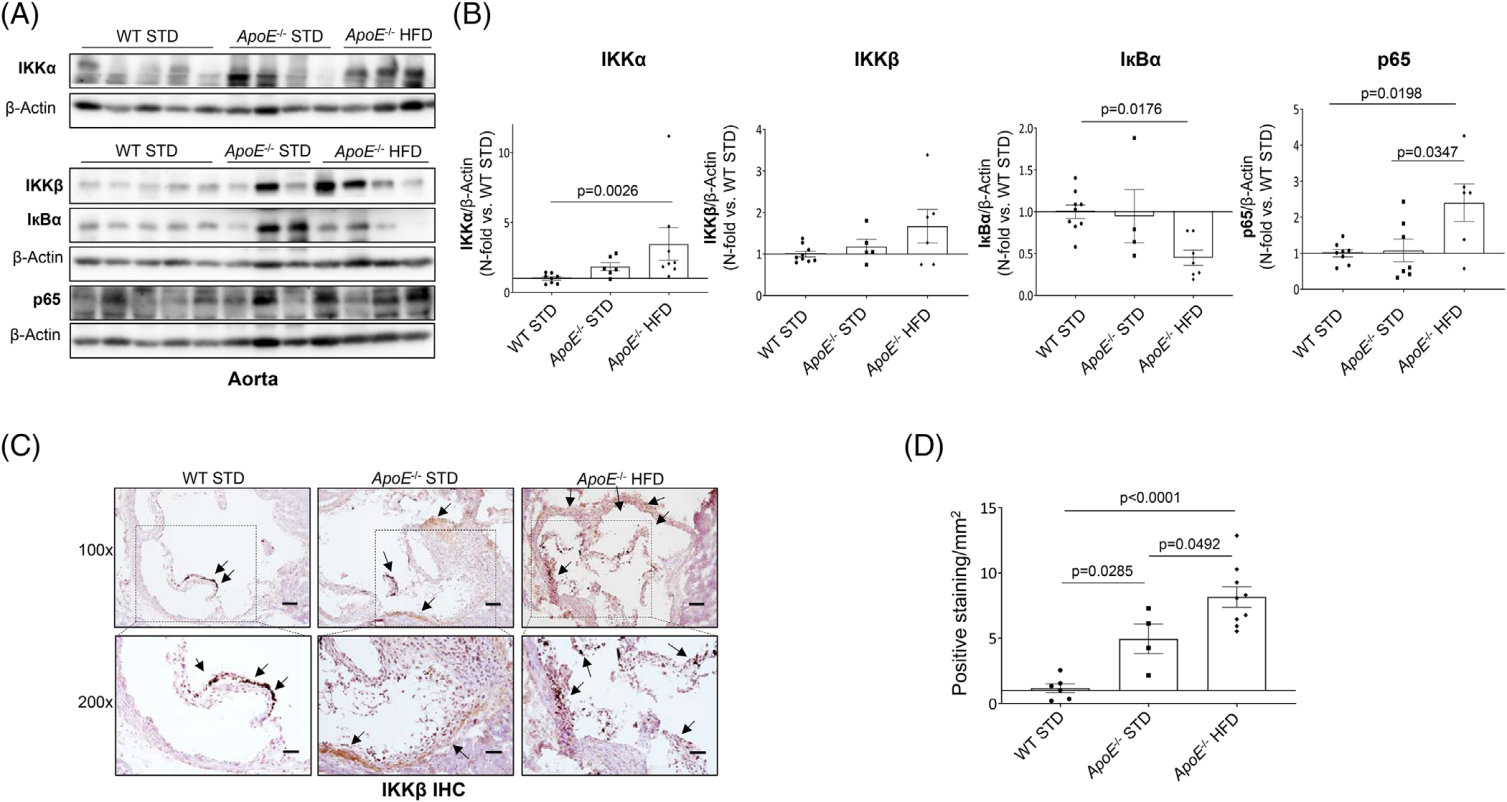
The expression of IKKα, IKKβ and p65 is upregulated in the aorta from *ApoE* ^−/−^ mice by HFD. (A) Representative Western blot images from the aorta of the three groups. (B) Measurement of the levels of IKKα, IKKβ, IкBα and p65 by Western blot in the aorta of the experimental groups submitted to 18 weeks of diet. (C) Representative images from the immunohistochemistry of IKKβ in the aortic root of the experimental groups. Magnification 100× (scale bar = 100 μm); magnification 200× (scale bar = 50 μm). (D) IKKβ expression was measured by immunohistochemistry. *ApoE* ^−/−^, *ApoE* deficient mice; HFD, high‐fat diet; IHC, immunohistochemistry; IкBα, nuclear factor kappa‐B kinase inhibitor alpha; IKKα, inhibitor of kappa‐B kinase subunit alpha; IKKβ, inhibitor of kappa‐B kinase subunit beta; p65, transcription factor p65; STD, standard type diet; WT, wild‐type group. Measurement of IKKα: WT STD (*n* = 8); *ApoE* ^−/−^ STD (*n* = 6); *ApoE* ^−/−^ HFD (*n* = 7). Measurement of IKKβ by Western blot: WT STD (*n* = 9); *ApoE* ^−/−^ STD (*n* = 5); *ApoE* ^−/−^ HFD (*n* = 6). Measurement of IкBα: WT STD (*n* = 9); *ApoE* ^−/−^ STD (*n* = 4); *ApoE* ^−/−^ HFD (*n* = 7). Measurement of p65: WT STD (*n* = 8); *ApoE* ^−/−^ STD (*n* = 7); *ApoE* ^−/−^ HFD (*n* = 6). Measurement of IKKβ by IHC: WT STD (*n* = 6); *ApoE* ^−/−^ STD (*n* = 4); *ApoE* ^−/−^ HFD (*n* = 9).

After that, by Western blot analysis, we demonstrated a significant increase in IKKα and p65 in aorta from *ApoE^−/−^* HFD mice and consequently a decrease of IκBα (Figure 3A,B). Moreover, by immunohistochemistry a significant increase of IKKβ was noted in aorta from both *ApoE^−/−^* mice, being clearly higher in *ApoE* ^−/−^ under HFD (Figure 3C,D). In Western blot studies the increase of IKKβ did not reach statistical significance (Figure 2A,B).

Moreover, we have demonstrated that p65 is expressed by endothelial cells and VSMCs in aortic roots, mainly in *ApoE* ^−/−^ HFD mice (Figures S6 and S7). More importantly, there is a strong increase of p65 in the nucleus, indicating NF‐κB activation. For these reasons, we have focused and deepened into the role of both miRNAs and its modulation in inflammation, specifically regarding NF‐κB activation through different in vitro experiments in HUVECs and VSMCs.

### 3.4 Overexpression of miR‐15a‐5p and miR‐199a‐3p reduced NF‐κB activation in endothelial cells

**FIGURE 4:**
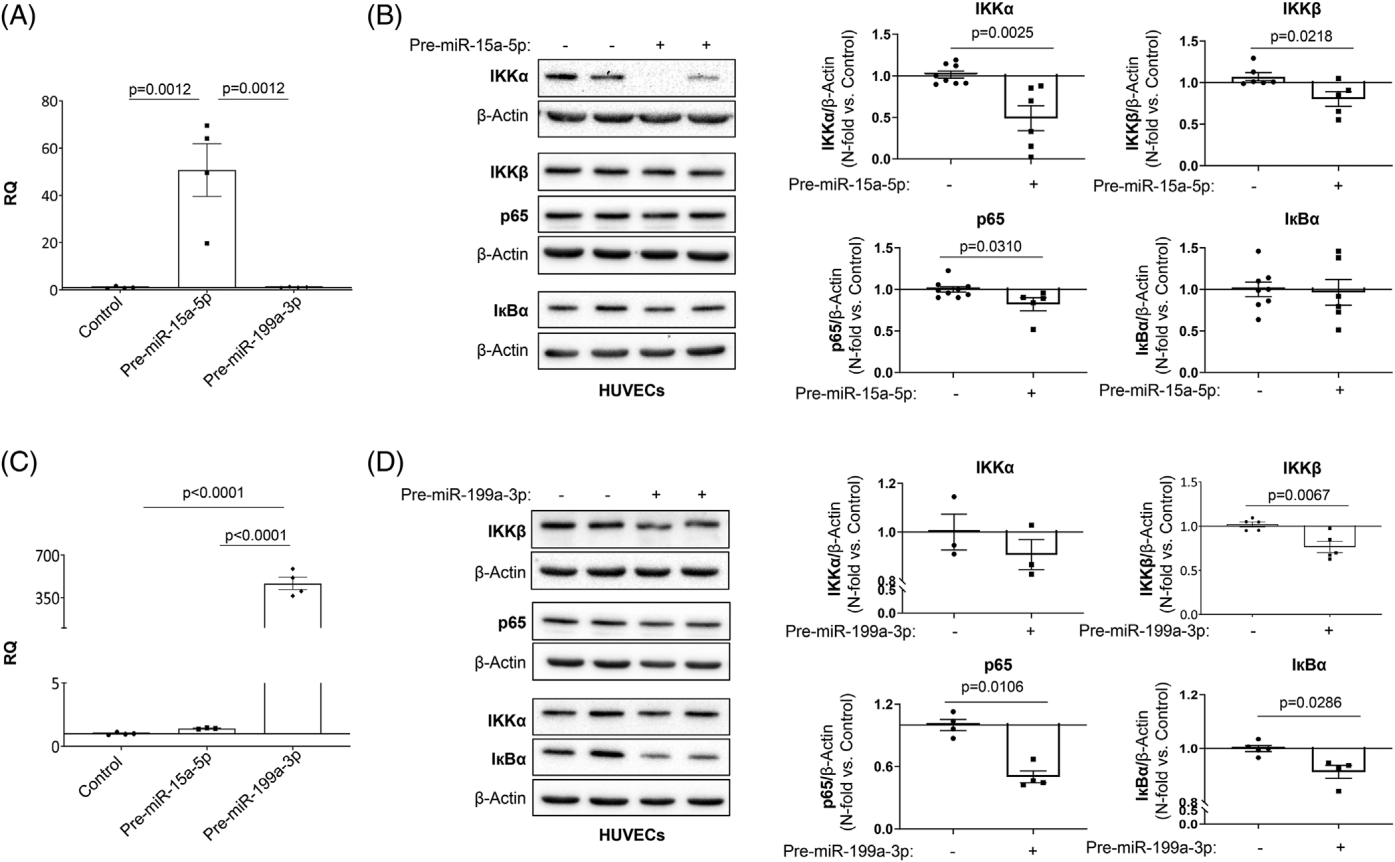
miR‐15a‐5p and miR‐199a‐3p overexpression reduced IKKα, IKKβ and p65 expression in endothelial cells. HUVECs were transfected with miR‐15a‐5p (A) or miR‐199a‐3p (C) precursors for 72 h. The increase in miRNA expression was measured by qPCR. The silencing effect miR‐15a‐5p or miR‐199a‐3p has on their targets IKKα (upper left) and IKKβ (upper right), as well as p65 (lower left) and IкBα (lower right), was analysed by Western blot 96 h after transfection (B and D, respectively). All the in vitro experiments were performed at least in triplicate. HUVECs, human umbilical vascular endothelial cells; IкBα, nuclear factor kappa‐B kinase inhibitor alpha; IKKα, inhibitor of nuclear factor kappa‐B kinase subunit alpha; IKKβ, inhibitor of nuclear factor kappa‐B kinase subunit beta; p65, transcription factor p65. qPCR miR‐15a‐5p: Control (*n* = 4); Pre‐miR‐15a‐5p (*n* = 4); Pre‐miR‐199a‐3p (*n* = 4). qPCR miR‐199a‐3p: Control (*n* = 4); Pre‐miR15a‐5p (*n* = 4); Pre‐miR‐199a‐3p (*n* = 4). Measurement of miR‐15a‐5p effect on IKKα: Control (*n* = 8), Pre‐miR‐15a‐5p (*n* = 6); effect on IKKβ: Control (*n* = 5), Pre‐miR‐15a‐5p (*n* = 5); effect on p65: control (*n* = 9); Pre‐miR‐15a‐5p (*n* = 5); effect on IкBα: control (*n* = 8); Pre‐miR‐15a‐5p (*n* = 6). Measurement of miR‐199a‐3p effect on IKKα: Control (*n* = 3), Pre‐miR‐199a‐3p (*n* = 3); effect on IKKβ: Control (*n* = 5), Pre‐miR‐199a‐3p (*n* = 5); effect on p65: Control (*n* = 4); pre‐miR‐199a‐3p (*n* = 4); effect on IкBα: Control (*n* = 5); pre‐miR‐199a‐3p (*n* = 4).

**FIGURE 5:**
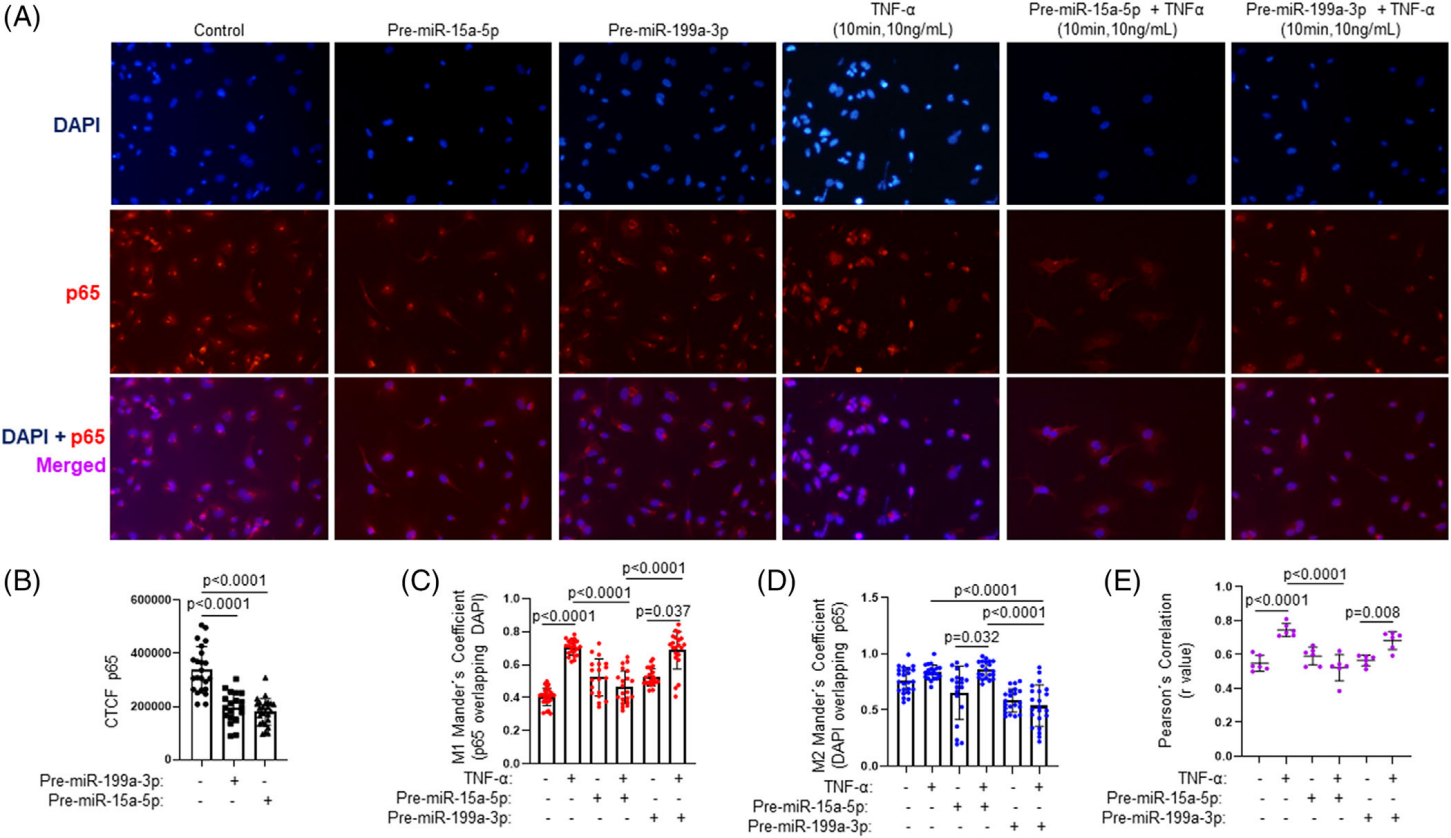
miR‐15a‐5p and miR‐199a‐3p reduced the activation of p65 in endothelial cells. HUVECs were stimulated with TNFα with or without pre‐miR‐15a‐5p, pre‐miR‐199a‐3p or both, and the activation of p65 was measured by immunofluorescence. (A) Representative images from the immunofluorescence of p65 (red) contrasted with DAPI (blue) and merged (purple). (B) The intensity of fluorescence of p65 was measured in all the conditions. Since p65 is active in the nucleus, the JaCoP plugin from Fiji was used to quantify the co‐localization of p65 and DAPI by three values: the M1 (C) and M2 (D) Mander's coefficient, and the Pearson's correlation (E) of each picture. In all cases, the closer the value is to 1, the more co‐localization. All the in vitro experiments were performed at least in triplicate. CTCF, corrected total cell fluorescence; p65, transcription factor p65; TNFα, tumour necrosis factor alpha. Measurement of p65 CTCF: Control (*n* = 23), Pre‐miR‐199a‐3p (*n* = 25); Pre‐miR‐15a‐5p (*n* = 19). Measurement of M1 Mander's coefficient: Control (*n* = 26), TNFα (*n* = 22), Pre‐miR‐199a‐3p (*n* = 24); Pre‐miR‐199a‐3p + TNFα (*n* = 21), Pre‐miR‐15a‐5p (*n* = 19); Pre‐miR‐15a‐5p + TNFα (*n* = 21). Measurement of M2 Mander's coefficient: Control (*n* = 24), TNFα (*n* = 21), Pre‐miR‐199a‐3p (*n* = 21); Pre‐miR‐199a‐3p + TNFα (*n* = 22), Pre‐miR‐15a‐5p (*n* = 20); Pre‐miR‐15a‐5p + TNFα (*n* = 21). Measurement of Pearson's correlation: Control (*n* = 6), TNFα (*n* = 6), Pre‐miR‐199a‐3p (*n* = 6); Pre‐miR‐199a‐3p + TNFα (*n* = 6), Pre‐miR‐15a‐5p (*n* = 6); Pre‐miR‐15a‐5p + TNFα (*n* = 6).

To explore whether the overexpression of miR‐15a‐5p and/or miR‐199a‐3p might reduce NF‐κB activation and consequently the inflammation present in the atherosclerotic process, HUVECs were transfected with the pre‐miR‐15a‐5p or pre‐miR‐199a‐3p. First, we confirmed that when HUVECs were transfected with pre‐miR‐15a‐5p or pre‐miR‐199a‐3p for 72 h, we obtained the overexpression of miR‐15a‐5p (Figure 4A) or miR‐199a‐3p (Figure 4C), respectively. After that, when we transfected with pre‐miR‐15a‐5p for 96 h, we observed a significant decrease in the protein levels of IKKα, IKKβ and p65 (Figure 4B). In the same way, when HUVECs were transfected with pre‐miR‐199a‐3p for 96 h, a significant reduction of IKKβ, p65 and IκBα was noted (Figure 4D). By immunofluorescence, overexpression of miR‐15a‐5p or miR‐199a‐3p significantly reduced p65 expression in HUVECs (Figure 5A,B).

Next, we analysed whether the overexpression of miR‐15a‐5p or miR‐199a‐3p was able to reduce NF‐κB activation. One of the mechanisms implicated in the activation of NF‐κB is the IKK complex activation by phosphorylation of IKKα and IKKβ. TNF‐α is widely used as an inductor of NF‐κB activation. For this reason, HUVECs were stimulated with 10 ng/mL TNF‐α at different times (10 to 40 min, see Figure S8) and we analysed the phosphorylation of IKK complex as well as the phosphorylation and translocation to the nucleus of p65 and the degradation of IκBα. So, we checked that 10 ng/mL TNF‐α for 10 min provoked the phosphorylation of IKKα/β (Figure S8A), a decrease of IκBα levels (Figure S8C,D), the phosphorylation and the translocation into the nucleus of p65 (Figure S8B,D). First, we confirmed that miR‐15a‐5p or miR‐199a‐3p overexpression significantly reduced the phosphorylation and the activation of IKKα/β induced by 10 ng/mL TNF‐α (Figure S9). After that, by immunofluorescence we also observed that miR‐15a‐5p or miR‐199a‐3p overexpression significantly decreased the translocation of p65 into the nucleus due to a lesser M1 and M2 Mander's coefficient (Figure 5A,C,D) as well as Pearson's Correlation (Figure 5A,E).

### 3.5 Overexpression of miR‐15a‐5p and miR‐199a‐3p reduced ox‐LDL uptake and NF‐κB activation in vascular smooth muscle cells

**FIGURE 6:**
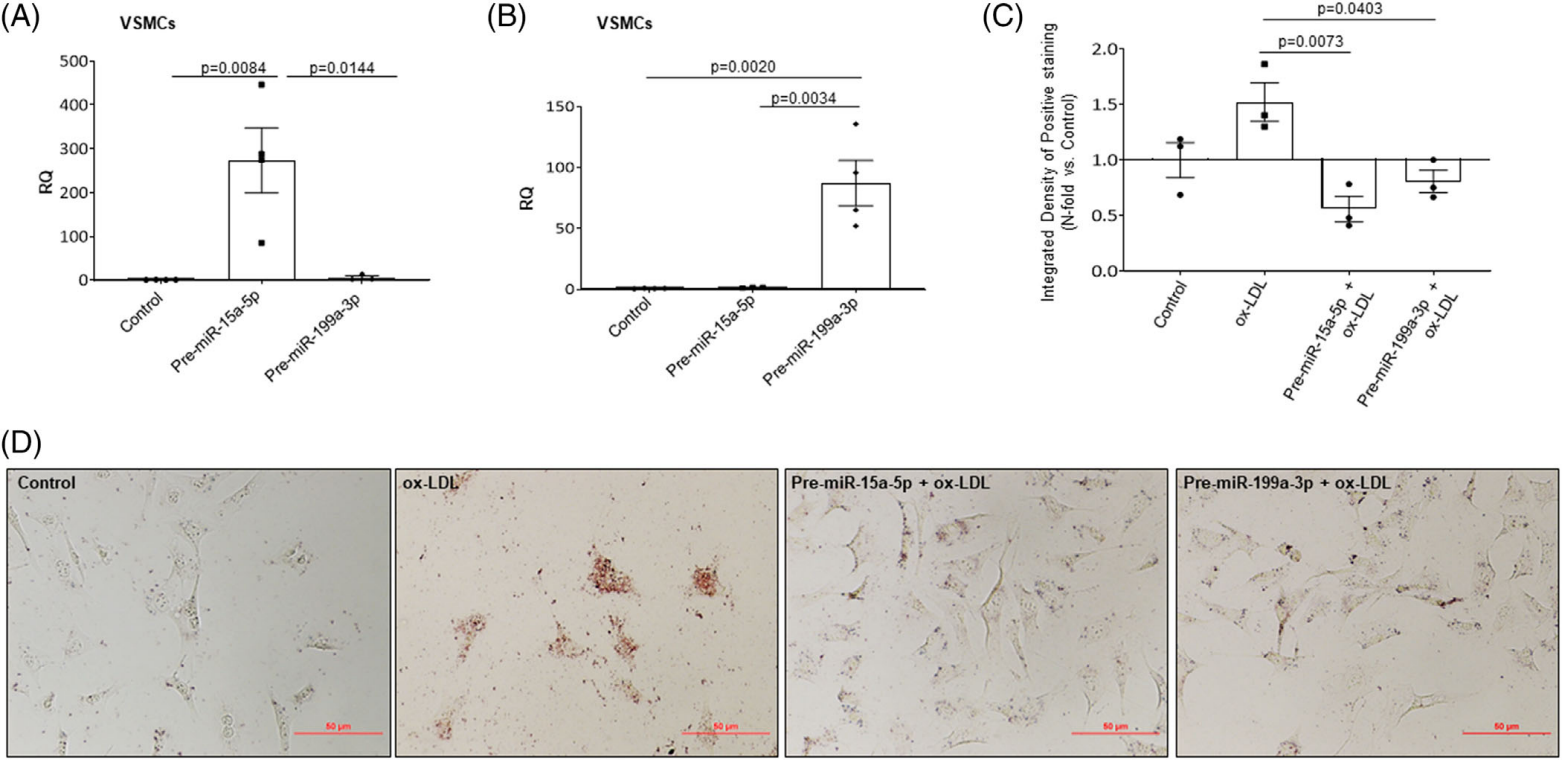
miR‐15a‐5p and miR‐199a‐3p overexpression reduced ox‐LDL uptake in VSMCs. VSMCs were transfected with miR‐15a‐5p (A) and miR199a‐3p (B) precursors for 48 h and the increase in miRNA expression was measured by qPCR. In the last 24 h of transfection, the cells were treated with ox‐LDL and then stained with Oil Red O. Quantification of measured the uptake of ox‐LDLs by VSMCs (C) and representative images from Oil Red O staining (D). All the in vitro experiments were performed at least in triplicate. ox‐LDL, oxidized low‐density lipoprotein; VSMCs, vascular smooth muscle cells. qPCR miR‐15a‐5p: Control (*n* = 4); Pre‐miR‐15a‐5p (*n* = 4); Pre‐miR‐199a‐3p (*n* = 3). qPCR miR‐199a‐3p: Control (*n* = 4); Pre‐miR‐15a‐5p (*n* = 3); Pre‐miR‐199a‐3p (*n* = 4). Measurement of ox‐LDL uptake: Control (*n* = 3), ox‐LDL (*n* = 3); Pre‐miR‐15a‐5p + ox‐LDL (*n* = 3); Pre‐miR‐199a‐3p + ox‐LDL (*n* = 3).

The next objective was to study whether the overexpression of miR‐15a‐5p or miR‐199a‐3p might interfere in LDL uptake and inflammation in VSMCs. For this aim, VSMCs were transfected for 72 h with pre‐miR‐15a‐5p or pre‐miR‐199a‐3p and we observed a significant increase of miR‐15a‐5p or miR‐199a‐3p by qRT‐PCR (Figures 6A and 6B, respectively). After that, we demonstrated that the overexpression of miR‐15a‐5p or miR‐199a‐3p reduced ox‐LDL uptake by VSMCs (Figure 6C). One of the mechanisms that might explain this result could be that miR‐15a‐5p or miR‐199a‐3p regulate LOX‐1 that is involved in ox‐LDL uptake by VSMCs. In this sense, we have shown a significant increase of LOX‐1 protein levels in ACA plaques (Figure S10A). Moreover, we found that LOX‐1 was expressed by VSMCs in human plaques and in aortic roots mainly from *ApoE* ^−/−^ HFD mice (Figures S10B and S11A, respectively). And finally, we demonstrate that miR‐15a‐5p overexpression significantly reduces LOX‐1 protein levels (Figure S11B).

**FIGURE 7:**
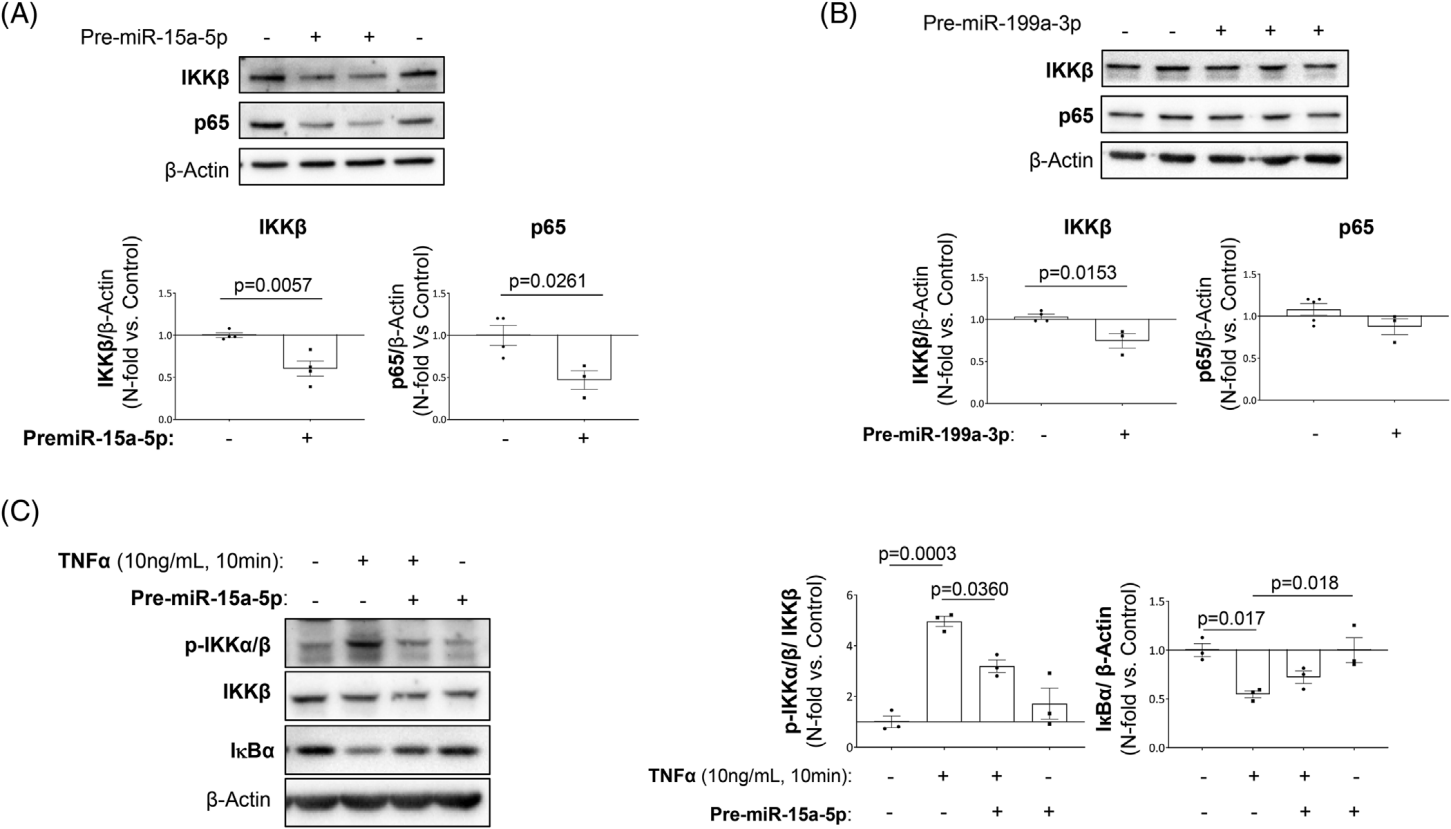
Effect of miR‐15a‐5p and miR‐199a‐3p overexpression in the expression of their targets in VSMCs. miR‐15a‐5p reduced IKKβ and p65 expression and IKKα and IKKβ activity while increasing IκBα, and miR‐199a‐3p reduced IKKβ expression in VSMCs. The silencing effect miR‐15a‐5p has on their targets (A) IKKβ (lower left), as well as p65 (lower right) was analysed by Western blot 48 h after transfection. The silencing effect miR‐199a‐3p has on their targets (B) IKKβ (lower left) and p65 (lower right) was analysed by Western blot 48 h after transfection. To test the effect on IKKα and IKKβ activity the VSMCs were pretreated with 0% FBS culture medium after the 48 h transfection and stimulated with TNFα for 10 min. (C) The activation of the proteins was analysed by the Western blot of their phosphorylated forms (left graph), and the expression of IκBα (right graph) was also analysed by Western blot. All the in vitro experiments were performed at least in triplicate. IкBα, nuclear factor kappa‐B kinase inhibitor alpha; IKKα, inhibitor of nuclear factor kappa‐B kinase subunit alpha; IKKβ, inhibitor of nuclear factor kappa‐B kinase subunit beta; p65, transcription factor p65; TNFα, tumour necrosis factor alpha. Measurement of miR‐15a‐5p effect on IKKβ: Control (*n* = 4), PremiR‐15a‐5p (*n* = 4), effect on p65: Control (*n* = 4); Pre‐miR‐15a‐5p (*n* = 3). Measurement of miR‐199a‐3p effect on IKKβ: Control (*n* = 4), Pre‐miR‐199a‐3p (*n* = 3). Measurement of miR‐199a‐3p effect on p65: Control (*n* = 5); Pre‐miR‐199a‐3p (*n* = 3). Measurement of miR‐15a‐5p effect on IKKβ and IKKα activity: Control (*n* = 3), TNFα (*n* = 3); Pre‐miR‐15a‐5p + TNFα (*n* = 3); Pre‐miR‐15a‐5p (*n* = 3). Measurement of miR‐15a‐5p effect on IкBα expression: Control (*n* = 3), TNFα (*n* = 3); Pre‐miR‐15a‐5p + TNFα (*n* = 3); Pre‐miR‐15a‐5p (*n* = 3).

Moreover, as occurs in HUVECs, we also confirmed in VSMCs that the overexpression of miR‐15a‐5p or miR‐199a‐3p reduced IKKβ and p65 protein levels (Figure 7A,B). In this sense, we found that the overexpression of miR‐15a‐5p significantly reduced the NF‐κB activation induced by TNF‐α (Figure 7C).

### 3.6 Regulation of IKBKB and CHUK expression through the direct interaction of miR‐15a‐5p or miR‐199a‐3p with their 3′UTR sequence

We performed experiments based on luciferase constructs to demonstrate the direct interaction of miR‐15a‐5p and miR‐199a‐3p with the 3ʹUTRs of p65, IKKβ and IKKα mRNAs (Figure S12). Our results demonstrate a direct and specific interaction of miR‐15a‐5p with the 3ʹUTR of IKBKB (IKKβ) and CHUK (IKKα) mRNAs and miR‐199a‐3p with the 3ʹUTR of IKBKB (IKKβ) mRNA. However, we did not find any change regarding the interaction of miR‐199a‐3p with the 3ʹUTR of RELA mRNA.

### 3.7 miR‐15a‐5p and miR‐199a‐3p as biomarkers of advanced atherosclerosis

**FIGURE 8:**
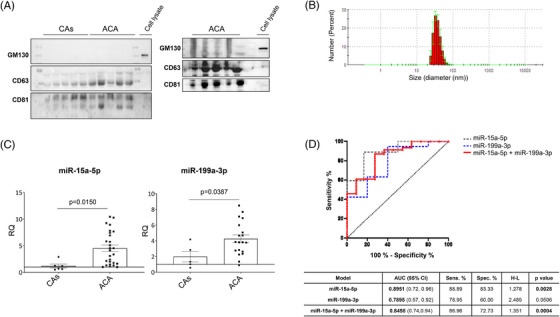
miR‐15a‐5p and miR‐199a‐3p are overexpressed in the plasmatic EVs of advance carotid atherosclerotic patients, and miR‐15‐5p may be a potential biomarker of the disease. To test whether miR‐15a‐5p and miR‐199a‐3p could be biomarkers of advanced atherosclerosis, we precipitated EVs from the plasma of healthy donors and advanced carotid atherosclerosis patients. Once the EVs were precipitated from the plasma, and in order to confirm that the pellet was enriched in exosomes, we performed the (A) Western blot of a negative exosomal biomarker, the Golgi membrane protein GM‐130; and two common markers from the exosomal membrane: CD63 and CD81, with a cell lysate as a control; and we also performed a DLS analysis of the samples (B) to confirm that the EVs were in the correct size range of exosomes. Afterwards the miRNAs were isolated from the exosomes and then the levels of miR‐15a‐5p and miR‐199a‐3p (C) were analysed by qPCR. The validity of the miR‐15a‐5p and miR‐199a‐3p (D) was confirmed by a ROC curve. ACA, advanced carotid atherosclerotic plaque patients; CAs, control subjects; ROC, receiver operating characteristic. qPCR of miR‐15a‐5p: CAs (*n* = 6), ACA (*n* = 27). qPCR of miR‐199a‐3p: CAs (*n* = 5); ACA (*n* = 19). ROC curve miR‐15a‐5p (*n* = 34) and ROC curve miR‐199a‐3p (*n* = 25).

Finally, trying to find novel miRNAs as possible diagnostic biomarkers for advanced atherosclerosis, we isolated miRNAs from plasma extracellular vesicles (EVs) from patients with ACA and Controls without atherosclerosis (see Table S1). To confirm whether the isolated EVs from plasma could be enriched in exosomes, we analysed the presence of the exosome markers CD63 antigen and CD81 antigen and the absence of Golgi subfamily A member 2 (GM130), a Golgi vesicle marker. We did not find GM130 in the isolated EVs compared to a positive control (HUVECs lysate) but we did find CD63 and CD81 expression (Figure 8A). In addition, we obtained that the diameter of the isolated EVs measured by DLS was between 10 and 106 nm, most exosomes having a diameter of 21.04–50.8 nm (Figure 8B). These findings support that the isolated EVs were enriched in exosomes.

Regarding circulating miRNA expression, we observed a significant increase of miR‐15a‐5p and miR‐199a‐3p (Figure 8C) in patients with ACA in relation to CAs. To evaluate their putative role as a diagnostic biomarker in advanced atherosclerosis, we performed ROC analyses (Figure 8D). For miR‐15a‐5p, the area under the curve was 0.8951 with a *p* value of 0.0028. The optimal cut‐off value for advanced atherosclerosis diagnosis was an RQ of 1.278 or higher, with a sensitivity of 88.89% and a specificity of 83.33% (Figure 8C). For miR‐199a‐3p, the area under the curve was 0.7895 with a *p* value of .0506 (Figure 8D). Finally, when we performed ROC analysis with both miRNAs simultaneously, the *p* value improved (*p* = .0004), although the sensitivity and the specificity were similar as compared with ROC analysis with only miR‐15a‐5p (Figure 8D).

## 4 DISCUSSION

Atherosclerosis is a disease that develops over several decades of life in an asymptomatic way, but when it progresses, it becomes one of the main causes of cardiovascular mortality, causing acute coronary syndrome or stroke.^21^ The development of acute events is mostly produced not by the complete stenosis of the vessel but because stable atherosclerotic plaques become unstable, favouring their rupture and the formation of a thrombus that can be occlusive and generate ischemia in that artery.^22^

For these reasons, it is of great interest to develop new strategies that allow the clinician to identify the presence of vulnerable plaques. More importantly, the identification of new markers or panels of new markers could make it possible to detect the change from stable to unstable plaques and the consequent increase in cardiovascular risk. In this context, the identification of miRNAs as disease mediators and new biomarkers are being widely studied in different metabolic diseases and could also be very useful in CVDs.^23^, ^24^ In this work, we have focused on four miRNAs, miR‐9‐5p, miR‐15a‐5p, miR‐16‐5p and miR‐199a‐3p, which we have considered of special relevance due to their involvement in inflammation,^18^ being one of the key events in the progression and instability of the atherosclerotic plaques. An increased presence of inflammatory cells, such as M1 macrophages, T lymphocytes, dendritic cells and eosinophils, has been found in symptomatic patients and in the vulnerable shoulder area of human carotid plaques.^25^, ^26^

From the four miRNAs studied, the most consistent results between human and mice atherosclerosis were for miR‐15a‐5p and miR‐199a‐3p. In human atherosclerosis, significant decreases of miR‐15a‐5p and miR‐199a‐3p were observed in advanced human atherosclerotic carotid plaques. Similarly, other authors had previously demonstrated a downregulation of miR‐199a/b‐3p in human atherosclerotic coronary arteries.^27^ In the experimental atherosclerosis model, we also found reduced levels of miR‐15a‐5p and miR‐199a‐3p in the aorta of *ApoE* ^−/−^ STD or HFD. In this sense, other study revealed that the levels of miR‐199a‐3p were diminished in aortas from mice with atherosclerosis.^28^ It should be noted that both miR‐15a‐5p and miR‐199a‐3p decreases were significantly higher as the disease progressed. Thus, the greatest decreases were found in the carotids from patients with advanced atherosclerosis and in the aorta from *ApoE* ^−/−^ mice subjected to HFD for 18 weeks. Moreover, in the experimental model we established a significant and inverse correlation between the levels of both miRNAs and percentage of lesion area and percentage of lipid depot in aortic roots. These results suggest the involvement of both miRNAs in the progression of atherosclerosis. In this sense, it has been described that serum levels of miR‐199a‐3p negatively correlated with the carotid intima‐media thickness and C reactive protein in patients with atherosclerosis.^29^ In addition, miR‐199a‐3p levels were decreased in the peripheral blood of T2DM patients and were associated with the progression of the disease.^30^

**FIGURE 9:**
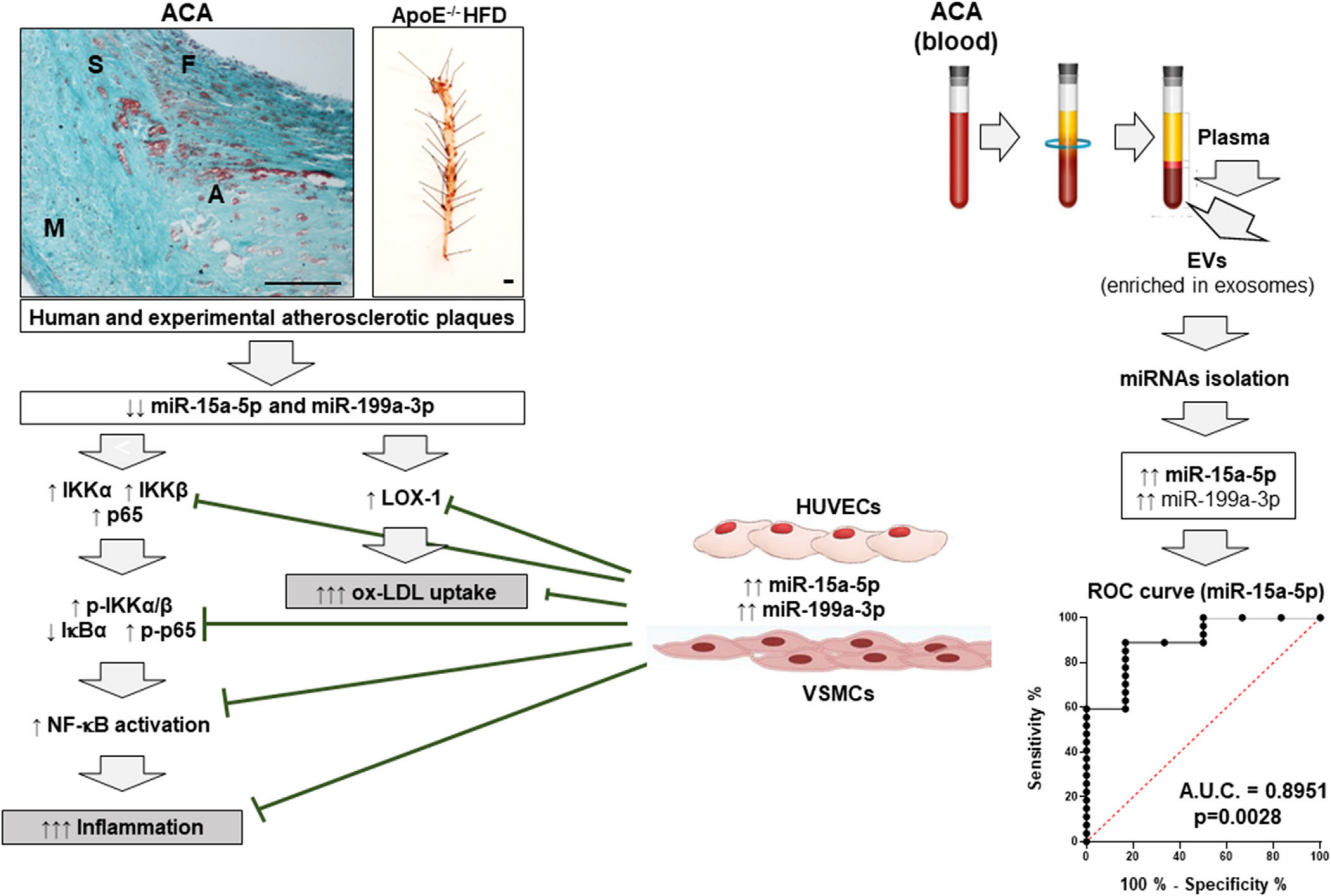
Role of miR‐15a‐5p and miR‐199a‐3p in the human and experimental atherosclerosis. The levels of miR‐15a‐5p and miR‐199a‐3p were significantly increased in carotid from patients with advanced atherosclerosis (ACA) and in the aortas from *ApoE* ^−/−^ HFD. These decreases of both miRNAs provoke a significant increase of their potential targets’ expression, such as: IKKα, IKKβ and p65 as well as their phosphorylation in consequence a higher IκBα degradation and NF‐κB activation, contributing to the inflammation present in advanced atherosclerosis. On the other hand, the decrease of miR‐15a‐5p and miR‐199a‐3p produce a significant increase of LOX‐1 that might favour ox‐LDL uptake in VSMCs. More importantly, in vascular cell lines, HUVECs and VSMCs, we demonstrated that miR‐15a‐5p or miR‐199a‐3p overexpression reduced protein levels of their studied targets in consequence the decline of NF‐κB activation and ox‐LDL uptake. Moreover, we isolated miRNAs from EVs (enriched exosomes) of patients with advanced atherosclerosis and healthy controls and a significant increase of both miRNAs in EVs from ACA was noted, whereas only miR‐15a‐5p might be useful as biomarker of advanced atherosclerosis.

There is a vast number of potential target genes regulated by miR‐15a‐5p or/and miR‐199a‐3p, which are involved in atherosclerosis, lipid metabolism and inflammation, including mTOR,^31^ ET‐1,^27^ YAP,^32^ CXCL11,^33^ CD44,^34^ MAP3K4,^35^ p65,^36^ IKKα/β,^37^ ICAM‐1^38^ and p85α.^39^ In addition to review of the literature, we performed in silico studies to determine possible targets of both miRNAs related to the inflammation, specifically, with proteins involved in NF‐κB pathway. We found as possible candidates: IKKα, IKKβ and p65. First, we confirmed elevated levels of IKKα, IKKβ and p65 in the aorta from *ApoE* ^−/−^ HFD 18 weeks and in carotids from patients with ACA. In VSMCs and HUVECs, we confirmed that overexpression of miR‐15a‐5p and/or miR‐199a‐3p reduced the protein levels of IKKα, IKKβ and p65 as well as NF‐κB activation, evaluated as lower degradation of IκBα and translocation of p65 to the nucleus. Similar to our results, other authors have found that overexpression of miR‐199a‐3p suppresses NF‐κB signalling in cervical epithelial cells^40^ increasing the migration, proliferation and autophagy of HUVECs, potentially through the regulation of PI3K/AKT/NF‐κB pathway.^30^ On the other hand, the miR‐199a‐3p restrained inflammation by downregulating RUNX1 in macrophages^18^ and deactivated STAT3 signalling in macrophages under ox‐LDL treatment.^41^ All these results point to and reinforce the idea that miR‐199a‐3p could have a protective role in the vascular endothelium and in the reduction of inflammation in the progression of atherosclerosis. In addition, our results also demonstrate that miR‐15a‐5p could have a new atheroprotective role by decreasing NF‐κB activation (Figure 9).

Continuing with the idea of the protective role of miR‐199a‐3p in the development of atherosclerosis, there are other authors who describe the regulation of other targets that could be involved in the vascular dysfunction and angiogenesis. So, in human atherosclerotic coronary arteries other authors observed a significant upregulation of ET‐1 and downregulation of miR‐199a/b‐3p.^27^ Neovascularization as angiogenesis in atherosclerotic lesions is a key factor in plaque growth and instability.^42^ Therefore, in atherosclerotic regions, hypoxia and local inflammation may induce intraplaque angiogenesis through growth and angiogenic factors, lipoproteins, MMPs and oxidized lipids.^43^, ^44^ In this context, miR‐199a‐3p has been described as a hypoxia‐related miRNA and can induce angiogenesis.^45^ Moreover, miR‐199a‐3p was related with markers of angiogenesis including neuropilin‐1, angiogenin and galectin‐3.^46^

We also demonstrated that both miRNAs regulate the function of VSMCs. Thus, we observed a differential expression of miR‐15a‐5p or miR‐199a‐3p in patients with initial or advanced atherosclerosis. In fibrolipidic plaques, the levels of both miRNAs were very similar or slightly higher than controls whereas in advanced atherosclerosis, there was a significant decrease of both miRNAs. This would fit with the protective role of both miRNAs. In early stages, miRNAs could be overexpressed as an atheroprotective mechanism, regulating different targets involved in the migration and proliferation of VSMCs to reduce vessel stenosis. Thus, it has been described that miR‐199a‐3p overexpression inhibited VSMCs migration and proliferation mediated by its target, SP1.^29^ In the current manuscript, we have demonstrated that miR‐15a‐5p or miR‐199a‐3p overexpression reduces the levels of theirs targets, IKKβ and p65 as well as NF‐κB activation contributing to a significant reduction of the inflammation produced by VSMCs. Moreover, an important percentage of foam cells could come from VSMCs that have lost specific markers of their contractile function and have gained membrane receptors, having a phenotype similar to macrophages, which allows them to capture modified LDL.^47^ In this sense, it has been described that VSMCs contain LOX‐1 as an endogenous ox‐LDL receptor that mediates the NF‐κB activation.^48^ According to this, we have also found elevated LOX‐1 protein levels in atherosclerotic carotid plaques from patients with ACA and in the aortic roots from *ApoE* ^−/−^ HFD mice and even, a higher number of VSMCs expressed LOX‐1 in both samples.

In in silico studies, we have obtained that one of the targets of miR‐15a‐5p is LOX‐1 and on the other hand, miR‐15a‐5p or miR‐199a‐3p overexpression reduced ox‐LDL uptake in VSMCs. Therefore, we demonstrated that miR‐15a‐5p overexpression downregulate LOX‐1 protein levels and in consequence VSMCs could uptake lower ox‐LDL, having both miRNAs an anti‐foaming role in VSMCs. In the case of miR‐199a‐3p might be by the downregulation of other endogenous ox‐LDL receptors but this anti‐foaming role of miR‐199a‐3p have previously been described in macrophages.^28^

Our results indicate that circulating miR‐15a‐5p levels are higher in patients with ACA, and ROC analyses suggesting a potential role for advanced atherosclerosis non‐invasive diagnosis. In this sense, a previous study also showed high miR‐15a‐5p levels in plasma from patients with stable coronary artery disease (CAD) compared with controls.^49^ More importantly, other authors have established a positive correlation between miR‐15a‐5p and coronary necrotic core as marker of plaque vulnerability in plaques from patients with CAD, while the exercise intervention induced a regression of coronary plaque burden and normalized miR‐15a‐5p levels.^50^ Other authors have also demonstrated that elevated circulating levels of miR‐15a‐5p along with miR‐34a‐5p and miR‐374‐5p in patients with aneurysmal subarachnoid haemorrhage, are potential biomarkers in aneurysmal rupture.^51^ There are more studies in the literature that support that miR‐15a‐5p might also be a biomarker of coronary heart disease (CHD).^52^, ^53^ In one of them, the authors used direct S‐Poly(T)Plus method for CHD diagnosis and determined that miR‐15a‐5p and miR‐199a‐3p together to other 10 miRNAs could be used for diagnosis of CHD.^52^ In this sense, Vegter et al.^46^ demonstrated that miR‐199a‐3p levels are related to angiogenesis markers in patients with heart failure. Moreover, there was a correlation between miR‐199a‐3p and galectin‐3 in patients with acute heart failure that might be due to the importance of cardiac remodelling and fibrosis in heart failure.^54^ In the current work, we also obtained a significantly higher level of miR‐199a‐3p in patients with ACA in relation to controls, being the *p* value of ROC curve close to statistical significance, potentially related to the small number of human samples included in this analysis.

## 5 CONCLUSIONS

In summary, our results demonstrate a novel role for miR‐15a‐5p and miR‐199a‐3p in atherosclerosis progression. Both miRNAs have anti‐inflammatory role due to regulation of their targets involved NF‐κB pathway in addition of anti‐foaming role in VSMCs. Finally, miR‐15a‐5p might be useful for diagnosis of ACA.

## CONFLICT OF INTEREST STATEMENT

The authors declare no conflicts of interest.

## Supporting information

Supporting Information

Supporting Information

Supporting Information

Supporting Information

Supporting Information

Supporting Information

Supporting Information

Supporting Information

Supporting Information

Supporting Information

Supporting Information

Supporting Information

Supporting Information
