# Supplementary figures and images for: Role of miR‐15a‐5p and miR‐199a‐3p in the inflammatory pathway regulated by NF‐κB in experimental and human atherosclerosis
Source: Clin Transl Med. 2023 Aug 21;13(8):e1363. doi: 10.1002/ctm2.1363 (PMC10442475; doi:10.1002/ctm2.1363)

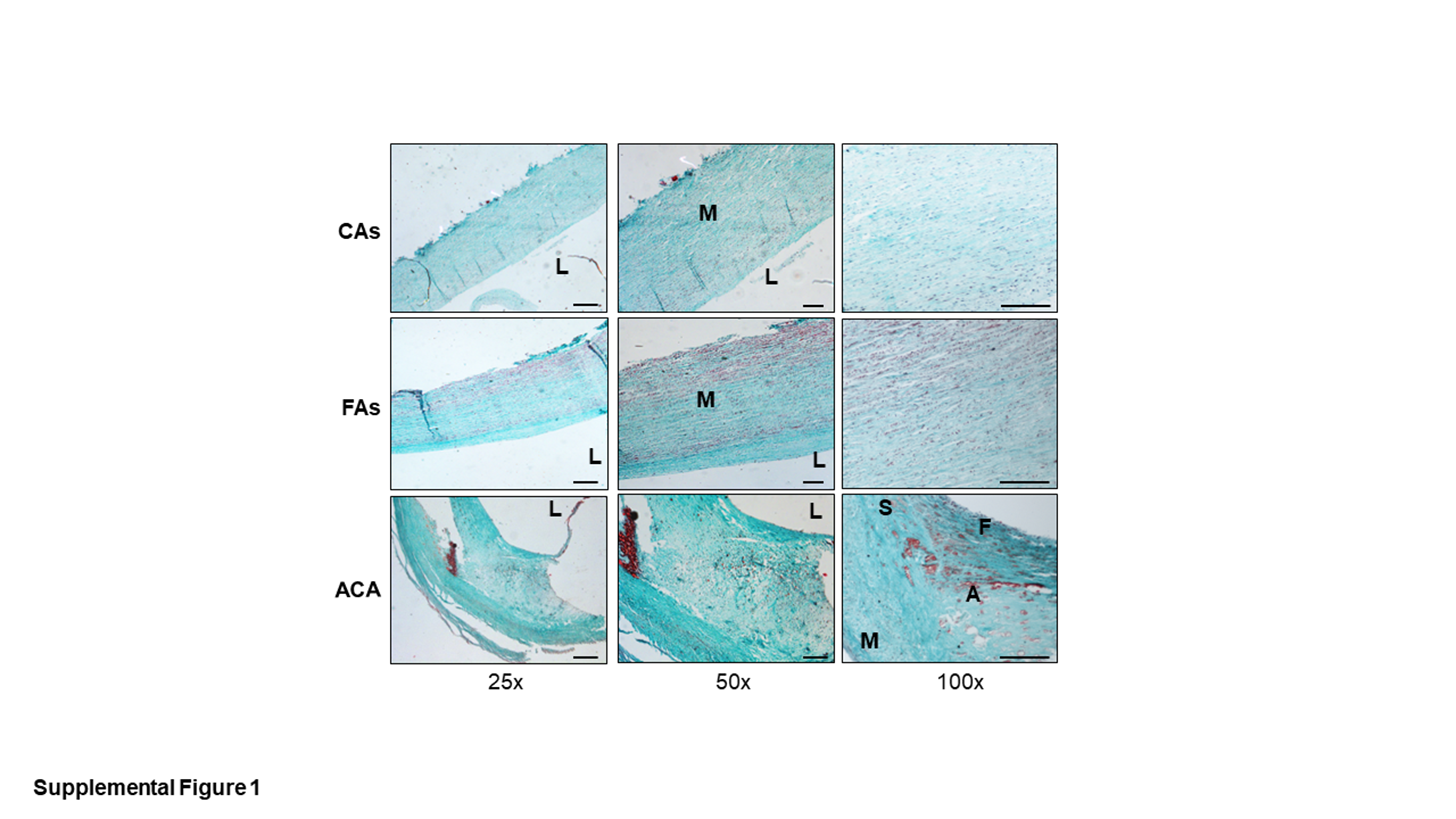

Supplement: Supplementary file 1 — Supporting Information [file CTM2-13-e1363-s001.TIF]

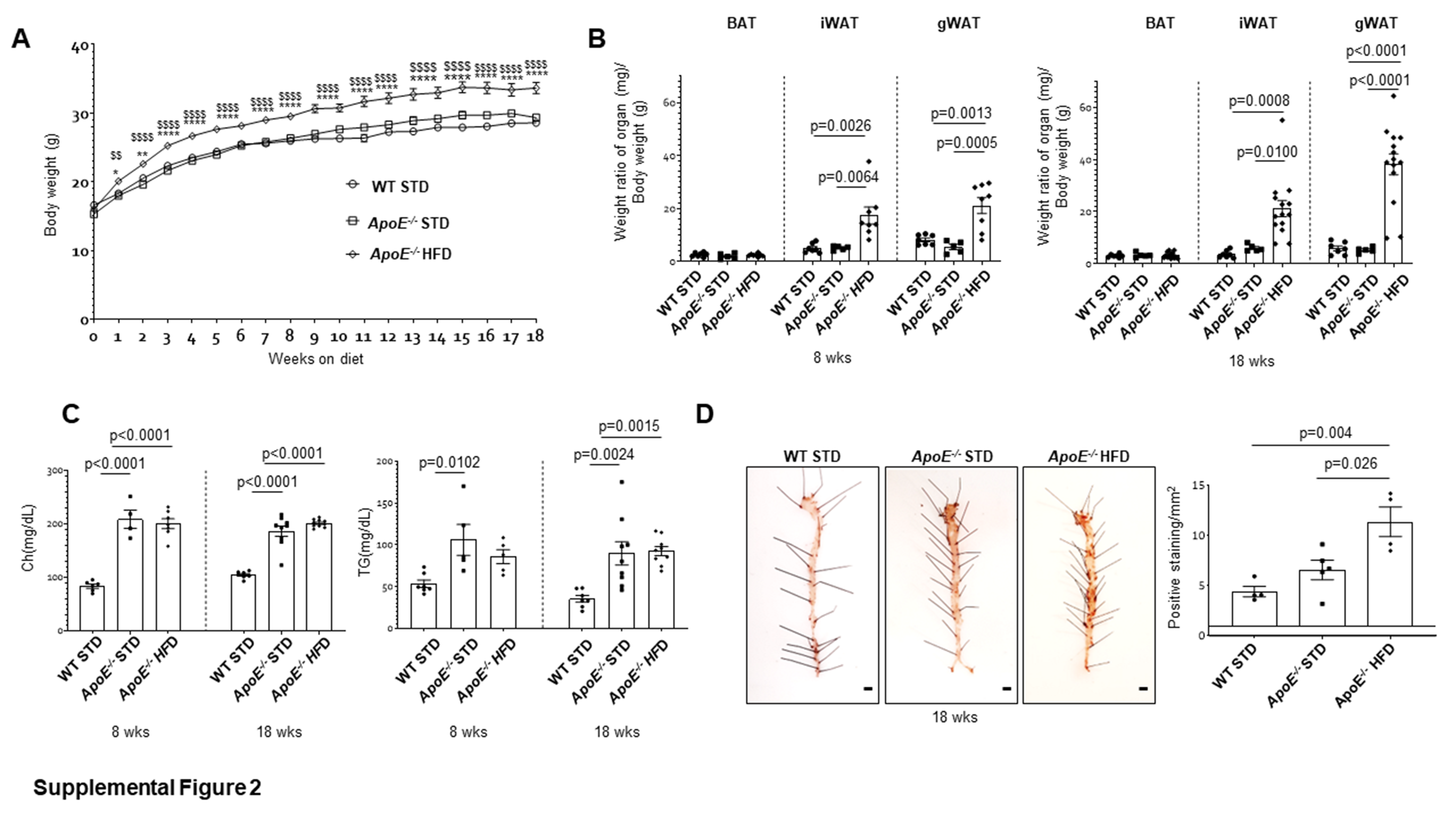

Supplement: Supplementary file 2 — Supporting Information [file CTM2-13-e1363-s012.TIF]

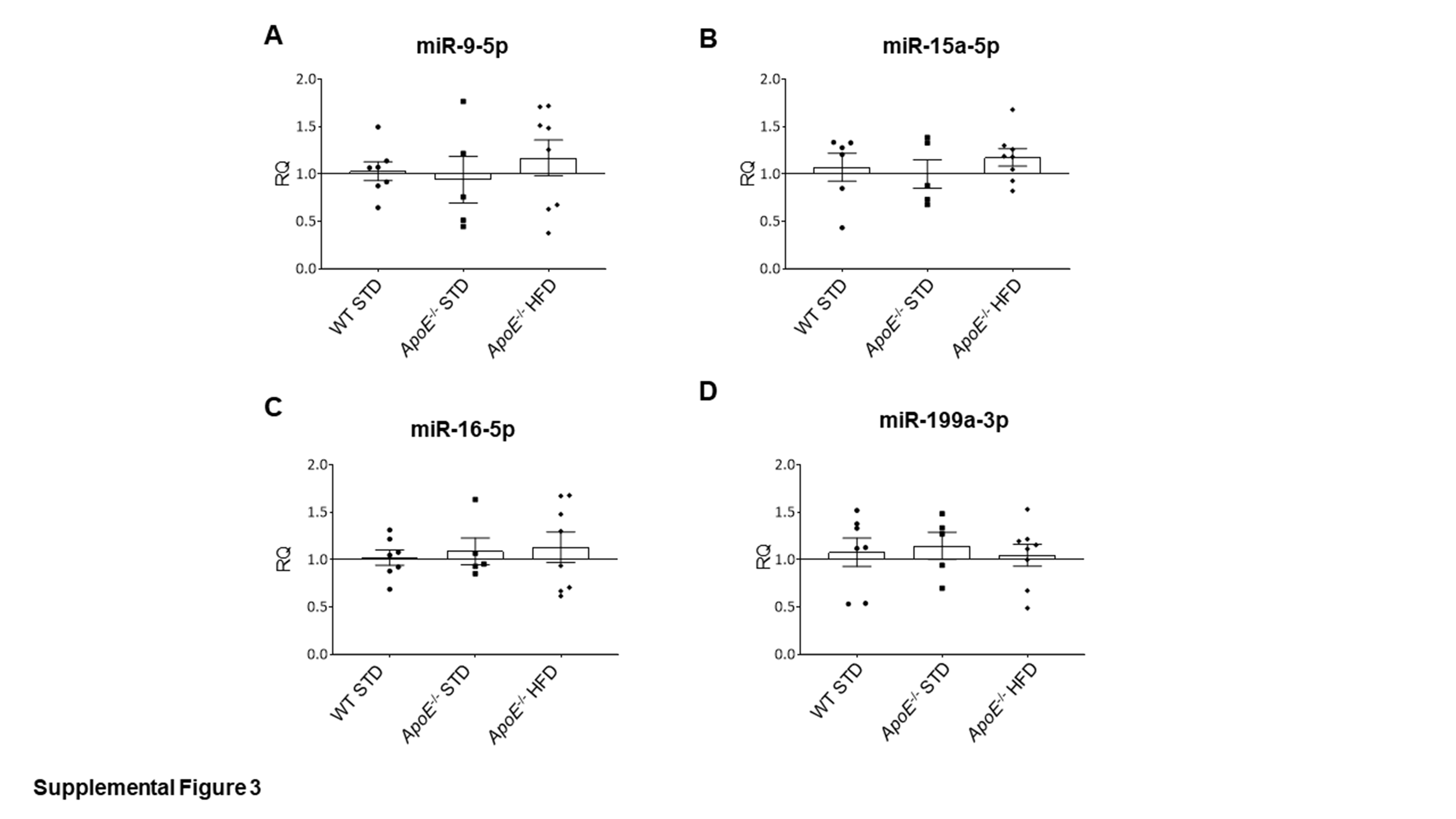

Supplement: Supplementary file 3 — Supporting Information [file CTM2-13-e1363-s006.TIF]

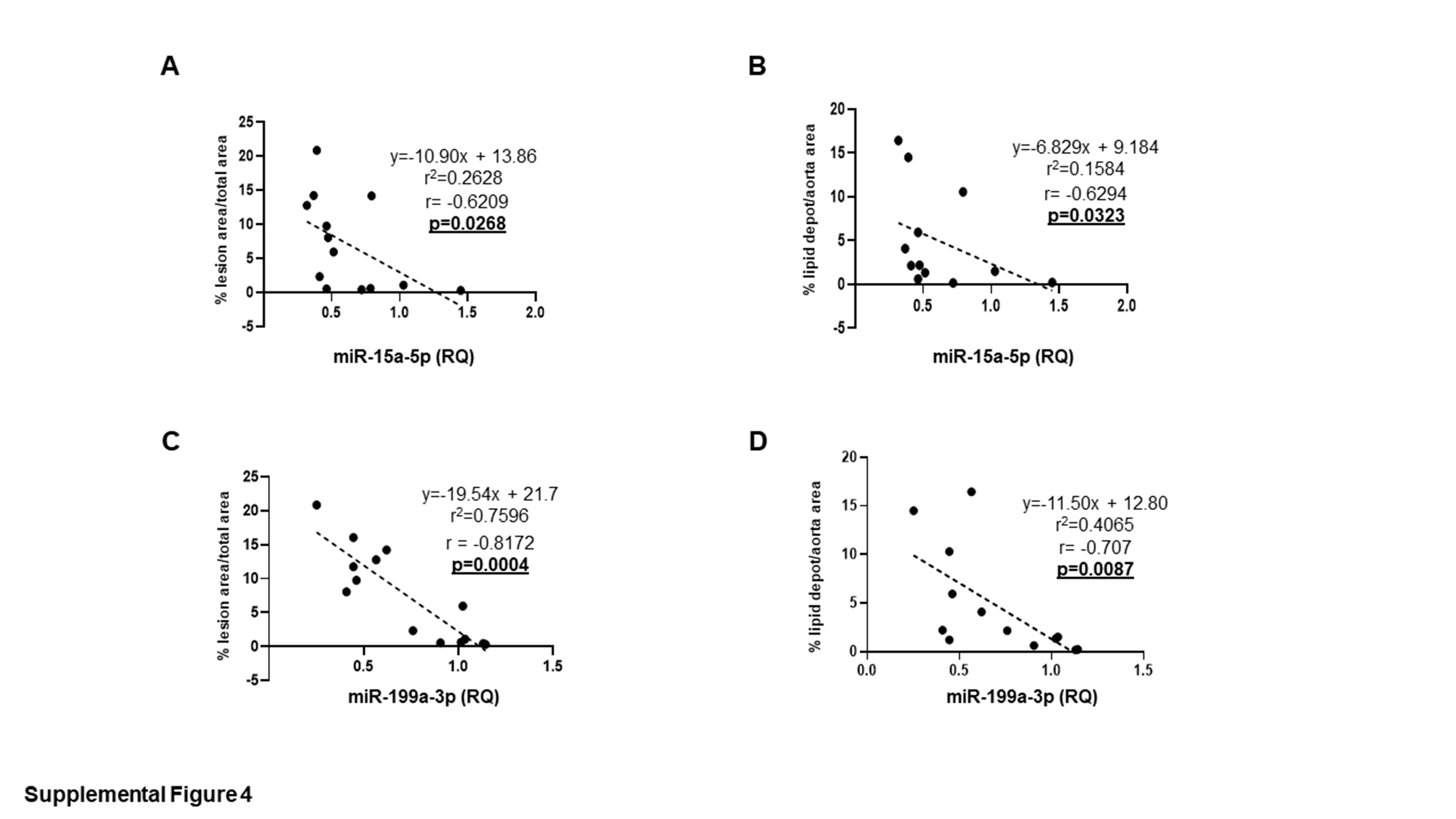

Supplement: Supplementary file 4 — Supporting Information [file CTM2-13-e1363-s013.TIF]

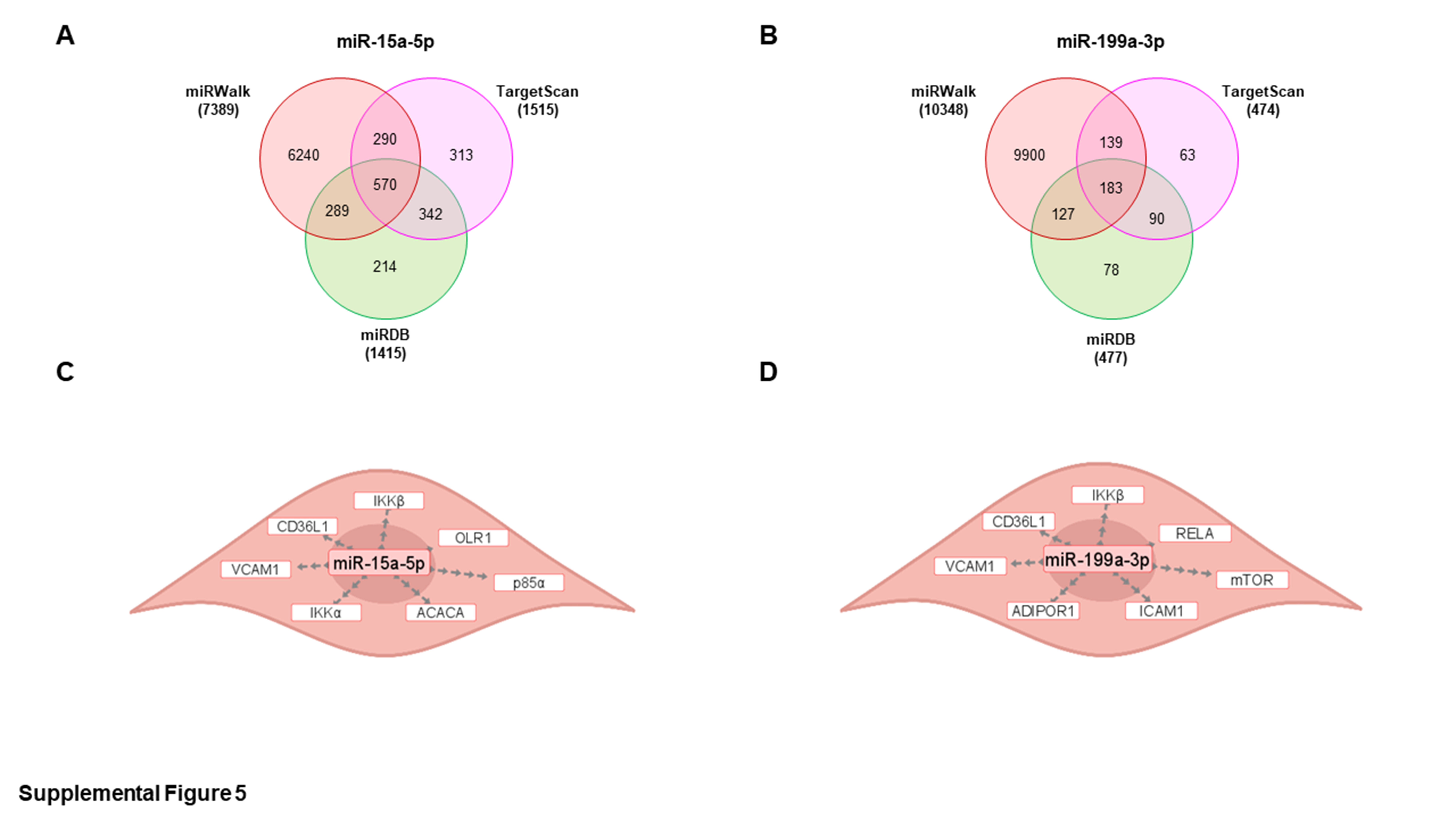

Supplement: Supplementary file 5 — Supporting Information [file CTM2-13-e1363-s002.TIF]

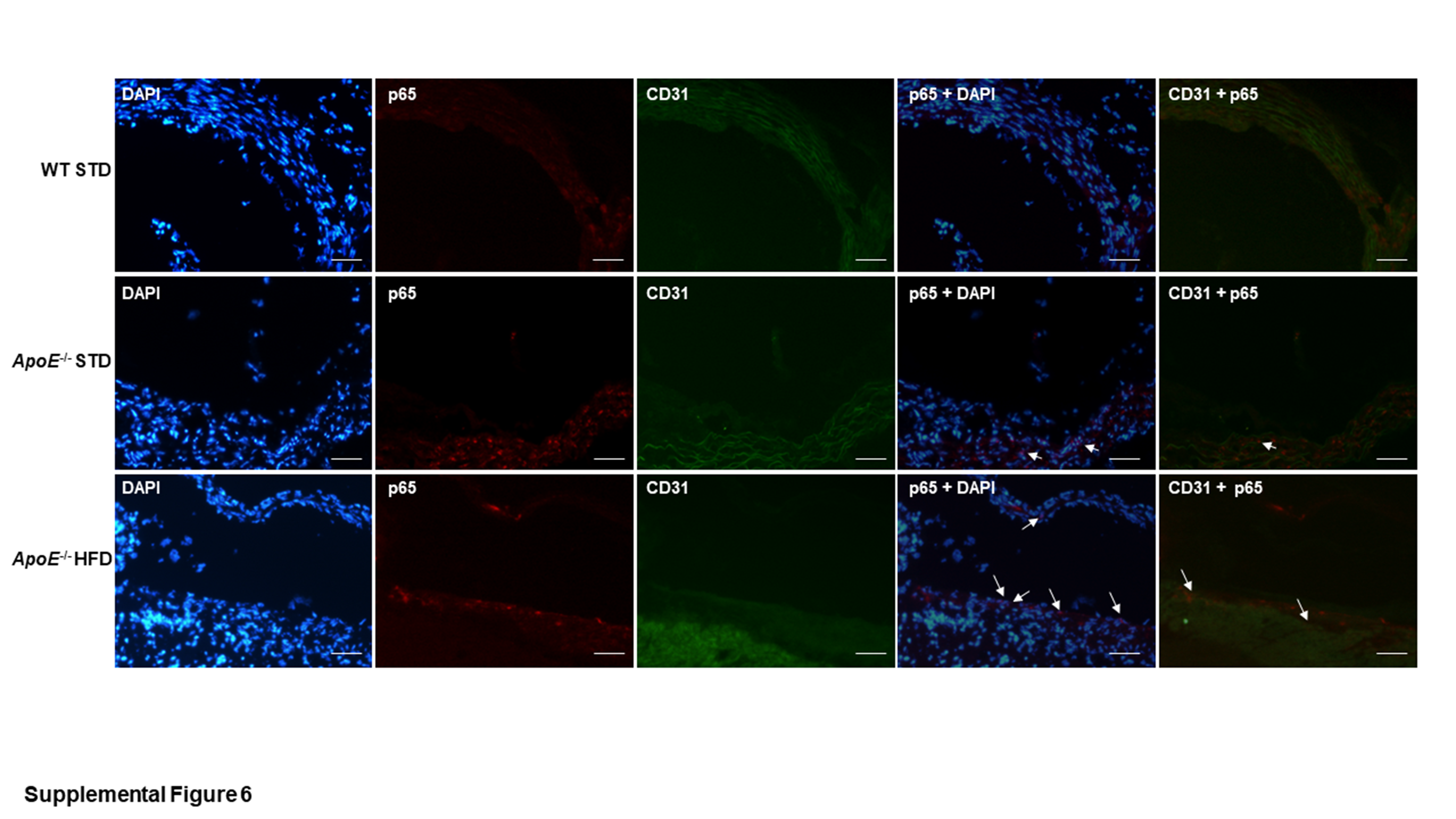

Supplement: Supplementary file 6 — Supporting Information [file CTM2-13-e1363-s009.TIF]

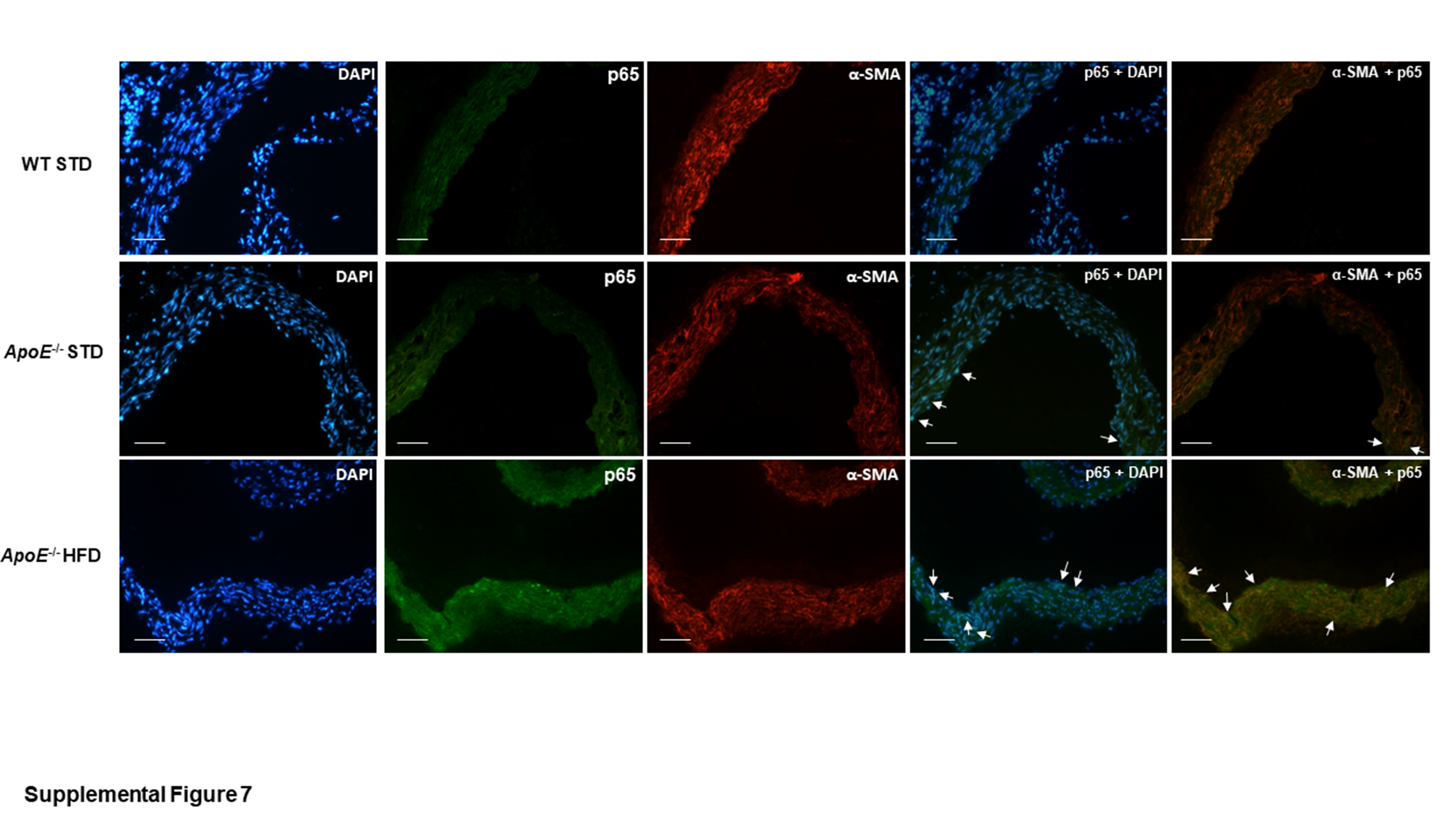

Supplement: Supplementary file 7 — Supporting Information [file CTM2-13-e1363-s005.TIF]

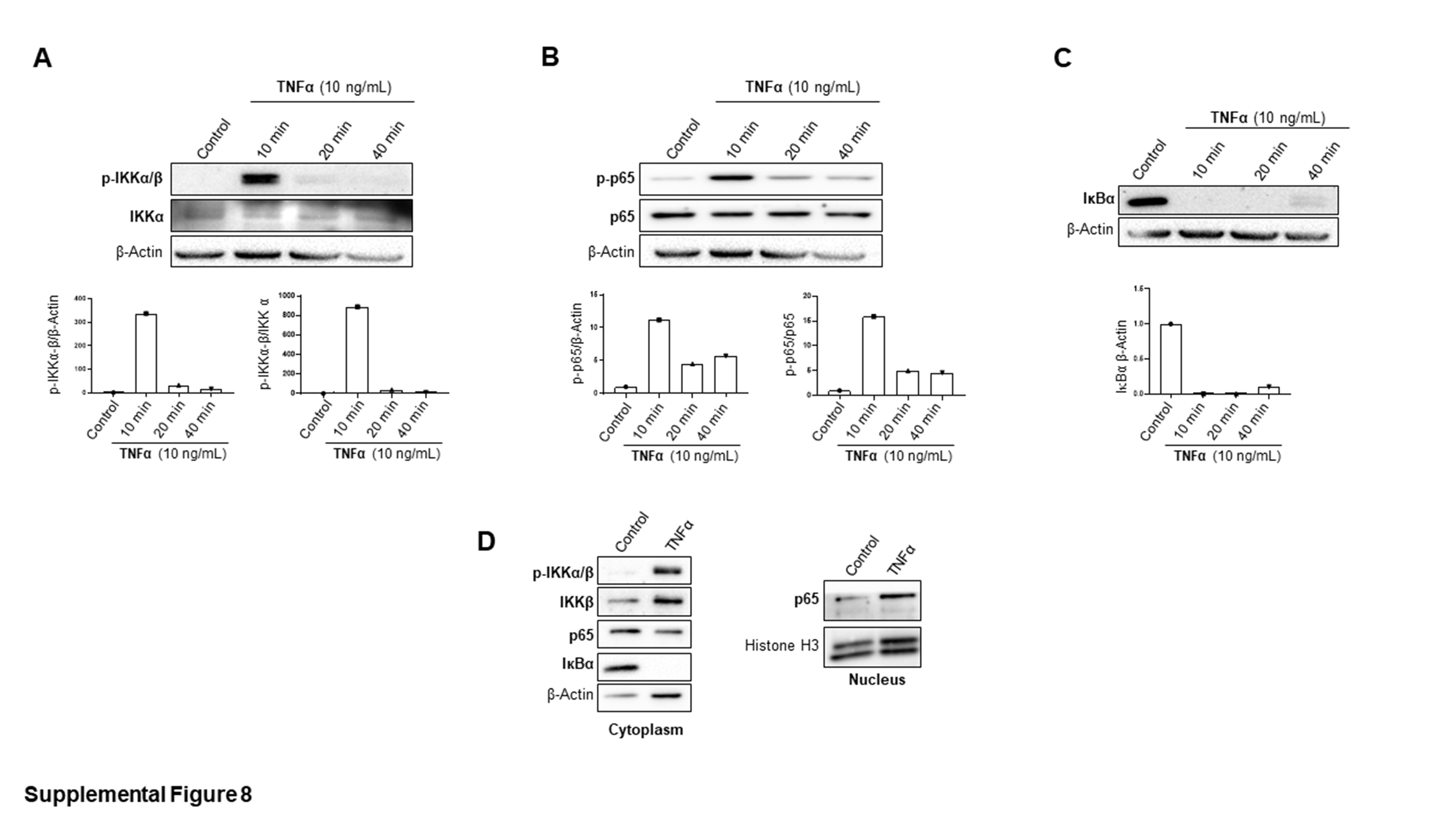

Supplement: Supplementary file 8 — Supporting Information [file CTM2-13-e1363-s008.TIF]

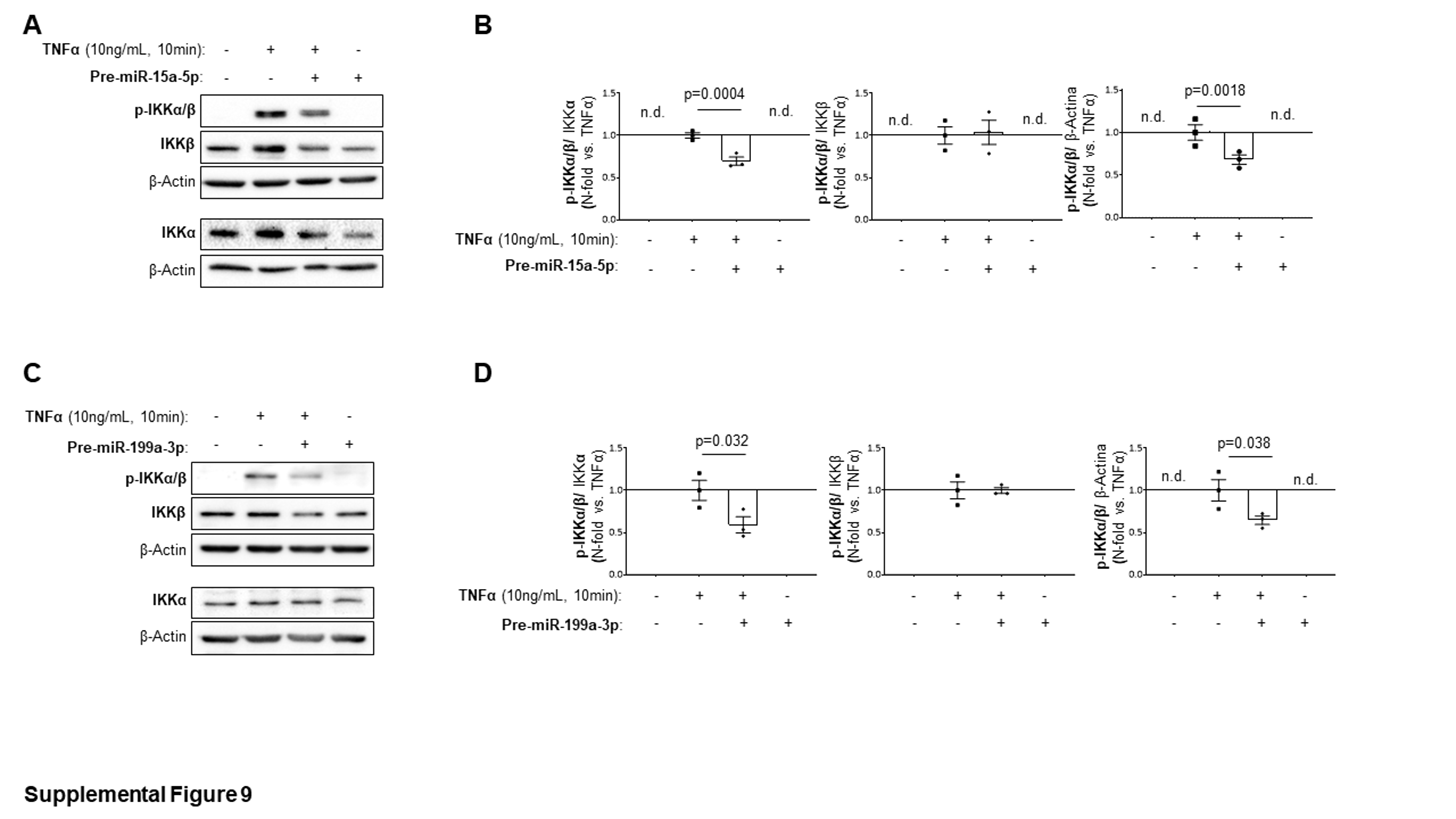

Supplement: Supplementary file 9 — Supporting Information [file CTM2-13-e1363-s011.TIF]

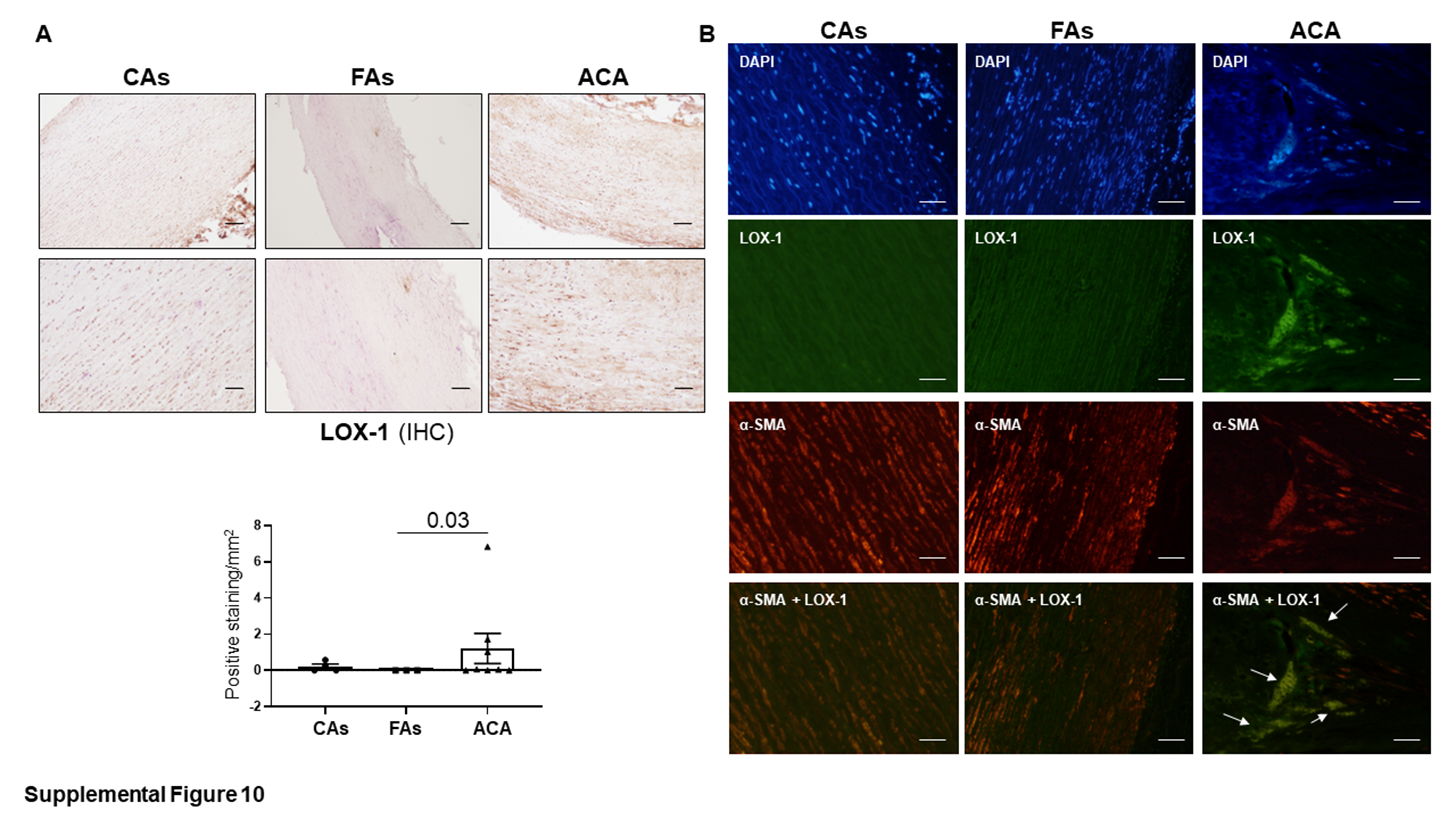

Supplement: Supplementary file 10 — Supporting Information [file CTM2-13-e1363-s003.TIF]

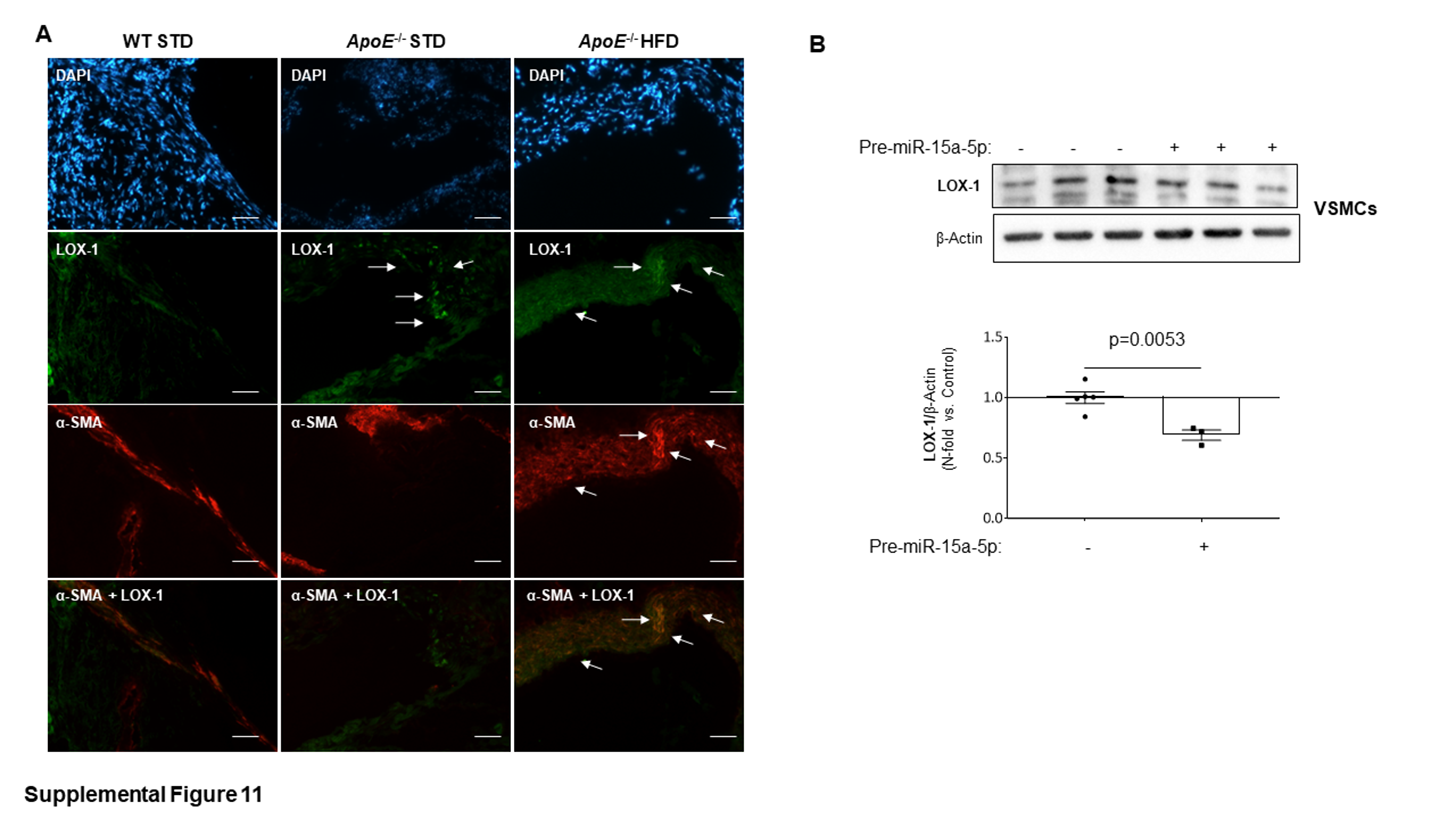

Supplement: Supplementary file 11 — Supporting Information [file CTM2-13-e1363-s004.TIF]

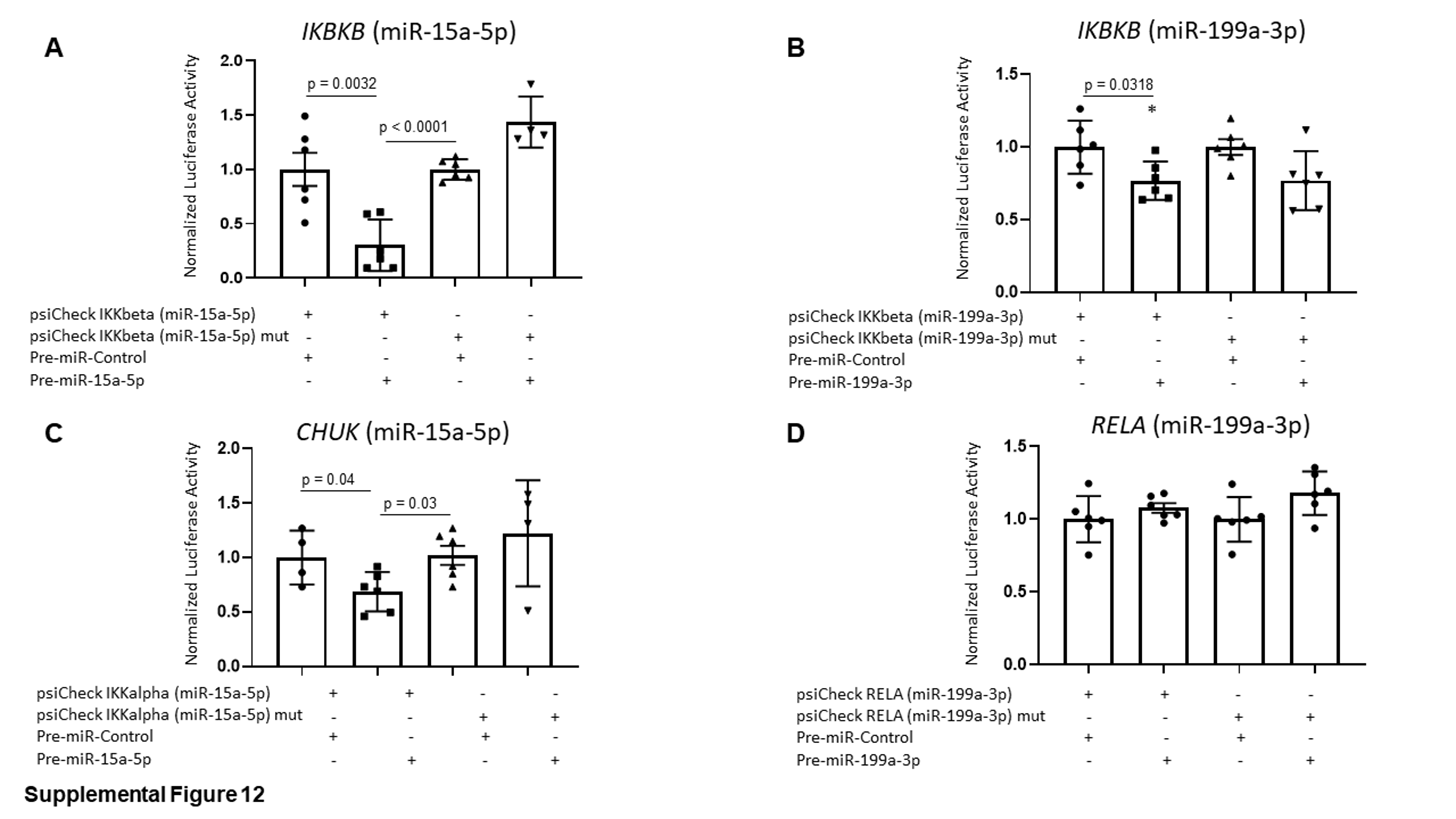

Supplement: Supplementary file 12 — Supporting Information [file CTM2-13-e1363-s007.TIF]
