# Supplementary material for: Role of miR‐15a‐5p and miR‐199a‐3p in the inflammatory pathway regulated by NF‐κB in experimental and human atherosclerosis
Source: Clin Transl Med. 2023 Aug 21;13(8):e1363. doi: 10.1002/ctm2.1363 (PMC10442475; doi:10.1002/ctm2.1363)
Supplement: Supplementary file 13 — Supporting Information [file CTM2-13-e1363-s010.docx]

**SUPPLEMENTAL DATA**

**SUpplementaL FIGURE LEGENDS**

**Supplemental Figure 1. Characterization of the atherosclerotic plaques.** To characterize the atherosclerotic human plaques, a Masson´s Trichromic staining was carried out. Representative images of the groups at a 25 (Scale bar = 200 µm), 50 (Scale bar = 100 µm) and 100 (Scale bar = 100 µm) zoom are shown. CAs= control subjects; FAs= subjects with fibrolipidic plaque; ACA= subjects with advanced carotid atherosclerotic plaque; M = media; F = fibrous; S = shoulder; A = atheroma; L = lumen.

**Supplemental Figure 2. Characterization of the experimental atherosclerosis model**. **(A)** The body weight of the different experimental groups was carried out since weaning up to sacrifice. **(B)** The weight of different adipose tissues was measured in the 8-week fed mice (left graph) and 18-week fed mice (right graph). **(C)** Measurement of cholesterol (left graph) and triglycerides (right graph) in fasted mice of the six groups of the experimental model. **(D)** *En face* staining of aortas from male C57BL/6 mice a fed STD, male ApoE^-/-^ mice fed a STD and male ApoE^-/-^ mice fed a HFD for 18 weeks. Scale bar = 100 μm**.** BAT= brown adipose tissue; iWAT= inguinal white adipose tissue; gWAT= gonadal white adipose tissue; Ch=cholesterol; TG=triglycerides; WT= Wild type group; STD= standard type diet; ApoE^-/-^ = ApoE deficient mice; HFD= high-fat diet; wks= weeks. 8 weeks BAT weight: WT STD (n=7); ApoE^-/-^ STD (n=5); ApoE^-/-^ HFD (n=8), iWAT weight: WT STD (n=7); ApoE^-/-^ STD (n=5); ApoE^-/-^ HFD (n=8), qWAT weight: WT STD (n=7); ApoE^-/-^ STD (n=5); ApoE^-/-^ HFD (n=8); 18 weeks BAT weight: WT STD (n=7); ApoE^-/-^ STD (n=5); ApoE^-/-^ HFD (n=14), iWAT weight: WT STD (n=7); ApoE^-/-^ STD (n=5); ApoE^-/-^ HFD (n=14), qWAT weight: WT STD (n=7); ApoE^-/-^ STD (n=5); ApoE^-/-^ HFD (n=14). 8 weeks Ch: WT STD (n=6), ApoE^-/-^ STD (n=4); ApoE^-/-^ HFD (n=7); 18 weeks Ch: WT STD (n=7), ApoE^-/-^ STD (n=9); ApoE^-/-^ HFD (n=10); 8 weeks TG: WT STD (n=7), ApoE^-/-^ STD (n=5); ApoE^-/-^ HFD (n=5); 18 weeks TG: WT STD (n=7), ApoE^-/-^ STD (n=9); ApoE^-/-^ HFD (n=9); *En face* analysis: WT STD (n=4), ApoE^-/-^ STD (n=5); ApoE^-/-^ HFD (n=4).

**Supplemental Figure 3. Study of the expression of miR-9-5p, miR-15a-5p, miR-16-5p and miR-199a-3p in the experimental atherosclerosis model.** The expression of miR-9-5p **(A)**, miR-15a-5p **(B)**, miR-16-5p **(C)** and miR-199a-3p **(D)** was analysed by RT-qPCR in the aorta of the mice-fed a STD or HFD for 8 weeks. RT-qPCR of miR-9-5p: WT STD (n=7), ApoE^-/-^ STD (n=5); ApoE^-/-^ HFD (n=8); qPCR of miR-15a-5p: WT STD (n=6), ApoE^-/-^ STD (n=5); ApoE^-/-^ HFD (n=8); RT-qPCR of miR-16-5p: WT STD (n=7), ApoE^-/-^ STD (n=5); ApoE^-/-^ HFD (n=8); qPCR of miR-199a-3p: WT STD (n=6), ApoE^-/-^ STD (n=5); ApoE^-/-^ HFD (n=8).

**Supplemental Figure 4. Correlation between miR-15a-5p and miR-199a-3p and the atherosclerosis progression.** By Spearman´s correlation, we set different correlation between miR-15a-5p and % area lesion/total area **(A)** and % lipid depot/ aorta area **(B)**; and miR-199a-3p % area lesion/total area **(C)** and % lipid depot/ aorta area **(D)** in mice of groups of 18 weeks of diet.

**Supplemental Figure 5. Possible targets stood out after *in silico* analysis for both miR-15a-5p and miR-199a-3p.** Venn diagrams with the number of targets from miRWalk, TargetScan and miRDB databases for the miR-15a-5p **(A)** and miR-199a-3p **(B)**. Schemes of a vascular smooth muscle cell with some interesting potential targets for miR-15a-5p **(C)** and miR-199a-3p **(D)**. IKKα= Inhibitor of kappa-B kinase subunit alpha; OLR1= Oxidized Low Density Lipoprotein Receptor 1; p85α= Phosphatidylinositol 3-kinase regulatory subunit alpha; ACACA= Acetyl-CoA carboxylase 1; VCAM1= Vascular cell adhesion protein 1; CD36L1= Scavenger Receptor Class B member 1; IKKβ= Inhibitor of kappa-B kinase subunit beta; IкBα= nuclear factor kappa-B kinase inhibitor alpha; RELA= transcription factor p65; mTOR= Mammalian target of rapamycin; ADIPOR1= Adiponectin receptor 1.

**Supplemental Figure 6. Expression of active p65 in endothelial cells from aortic roots in an experimental model of atherosclerosis**. In aortic roots from WT STD 18 wks, ApoE^-/-^ STD 18 wks and ApoE^-/-^ HFD 18 wks, we analyzed the colocalization of p65 (red) and DAPI (blue) to detect active p65 (p65 merged with DAPI). Moreover, we also colocalized p65 with CD31 (green) to detect endothelial cells that express p65 and active p65 (p65 merged with CD31). Magnification 200x, scale bar = 50 μm.

**Supplemental Figure 7. Expression of active p65 in vascular smooth muscle cells from aortic roots in an experimental model of atherosclerosis**. In aortic roots from WT STD 18 wks, ApoE^-/-^ STD 18 wks and ApoE^-/-^ HFD 18 wks, we analyzed the colocalization of p65 (green) and DAPI (blue) to detect active p65 (p65 merged with DAPI). Moreover, we also colocalized p65 with α-SMA to detect VSMCs that express p65 and active p65 (p65 merged with α-SMA). Magnification 200x, scale bar = 50 μm.

**Supplemental Figure 8. Effect of TNF-α in NF-κB pathway in HUVECs.** To establish the correct time of stimulation with TNFα, a time course was done and the activation of the pathway was assessed by Western blot of p-IKKα/β **(A)**, p-p65 **(B)** and IκBα **(C)** at 10, 20 and 40 minutes of stimulation with TNFα (10 ug/mL). The same proteins were studied by Western blot after a cellular sub-fractioning of cytoplasm and nucleus **(D)** to confirm that p65 was being translocated to the nucleus. IKKα= Inhibitor of nuclear factor kappa-B kinase subunit alpha; IKKβ= Inhibitor of nuclear factor kappa-B kinase subunit beta; IкBα= nuclear factor kappa-B kinase inhibitor alpha; p65 = transcription factor p65; TNFα= tumor necrosis factor alpha; min= minutes.

**Supplemental Figure 9. The effect of miR-15a-5p and miR-199a-3p in the activity of IKK complex.** HUVECs were treated with TNFα (10 ug/mL) for 10 minutes after the transfection with pre-miR-15a-5p or pre-miR-199a-3p and after that the protein levels of their targets were analyzed by Western blot **(A and C)** and their quantifications represented in different graphs **(B and D)**. MiR15a-5p or miR-199a-3p overexpression reduced the phosphorylation of IKKα/β. IKKα= Inhibitor of nuclear factor kappa-B kinase subunit alpha; IKKβ= Inhibitor of nuclear factor kappa-B kinase subunit beta; IкBα= nuclear factor kappa-B kinase inhibitor alpha; p65= transcription factor p65; TNFα= tumor necrosis factor alpha; min= minutes. All the *in vitro* experiments were performed at least in triplicate.

**Supplemental Figure 10. Role of LOX-1 in the progression of human atherosclerosis. (A)** We studied LOX-1 protein levels in aorta from CAs, FAs and in carotid from ACA patients by immunohistochemistry with LOX-1 antibody. The quantification is expressed as positive staining/mm^2^. Upper images (magnification 100x, scale bar = 50 μm), lower images (magnification 200X, scale bar = 50 μm). **(B)** Double immunofluorescence of LOX-1 (green) and α-SMA (red) in vascular samples of CAs, FAs and ACA. These results show that LOX-1 expression is increased in atherosclerotic carotid plaques of ACA patients. Magnification 200x, scale bar = 50 μm

**Supplemental Figure 11. Role of LOX-1 in the progression of experimental atherosclerosis. (A)** Double immunofluorescence of LOX-1 (green) and α-SMA (red) in aortic roots from WT STD 18 wks, ApoE^-/-^ STD 18 wks and ApoE^-/-^ HFD 18 wks. These results show that LOX-1 expression is increased in atherosclerotic plaques from ApoE^-/-^ HFD mice. Magnification 200X, scale bar = 50 μm. **(B)** VSMCs were transfected with Pre-miR-15a-5p for 48 hours and we measured LOX-1 protein levels by Western-blot. miR-15a-5p overexpression reduced LOX-1 protein levels. The experiment was performed 4 times.

**Supplemental Figure 12. Regulation of *IKBKB* and *CHUK* expression by the interaction of miR-15a-5p or miR-199a-3p with their 3′UTR sequence.** Shown is the normalized Renilla luciferase activity in HEK293 cells transfected with the different constructs. **(A)** HEK293 cells were co-transfected with the psiCHECK IKKbeta (miR-15a-5p) and psiCHECK IKKbeta (miR-15a-5p) mut plasmids together with Pre-miR control or Pre-miR-15a-5p. **(B)** HEK293 cells were co-transfected with the psiCHECK IKKbeta (miR-199a-3p) and psiCHECK IKKbeta (miR-199a-3p) mut plasmids together with Pre-miR control or Pre-miR-199a-3p. (**C)** HEK293 cells were co-transfected with the psiCHECK IKKalpha (miR-15a-5p) and psiCHECK IKKalpha (miR-15a-5p) mut plasmids together with Pre-miR control or Pre-miR-15a-5p. **(D)** HEK293 cells were co-transfected with the psiCHECK RELA (miR-199a-3p) and psiCHECK RELA (miR-199a-3p) mut plasmids together with Pre-miR control or Pre-miR-199a-3p. In all cases, Renilla luciferase activity was normalized to firefly luciferase activity. Bars show mean values ± SEM corresponding to 3-5 independent experiments.

**SUPPLEMENTAL TABLES**

**Supplemental Table 1. Clinical characteristics of patients bearing advanced carotid atherosclerosis**

|  | **Patients ACA**  **(Carotid Plaque) (n=14)** | **Patients ACA**  **(Exosomes)**  **(n=29)** |
| --- | --- | --- |
| **Age, years** | 70±7 | 67±10 |
| **Gender (male/female), %** | 71.4% / 28.6% | 72.4% / 27.6% |
| **BMI (Kg/m^2^)** | 29.2± 6.3 | 28.8 ± 4.8 |
| **Diabetes mellitus, %** | 42.9% | 34.50% |
| **Hypertension, %** | 85.7% | 75.80% |
| **Coronary artery disease, %** | 100% | 100% |
| **Current smoking, %** | 37.7% | 27.60% |

**Supplemental Table 2. Antibodies used in Western blot and immunohistochemistry studies.**

| **Antibody** | **Dilution** | **Supplier** | **Reference** | **Technique** |  |
| --- | --- | --- | --- | --- | --- |
| **p-IKKα/β (Ser176/180)** | 1:1000 | Cell Signalling Technology | #2697 | WB |  |
| **IKKα (B-8)** | 1:250 | Santa Cruz Biotechnology | sc-7606 | WB |  |
|  | 1:25 |  |  | IHC-P |  |
| **IKKβ** | 1:1000 | Cell Signalling Technology | #2678 | WB |  |
|  |  |  |  |  |  |
|  | 1:50 |  |  | IHC-P |  |
| **IKKβ** | 1:200 | Proteintech | 15649-1-AP | OCT-IHC |  |
| **IкBα (L35A5)** | 1:2000 | Cell Signalling Technology | #4814 | WB |  |
| **NF-кB p65** | 1:2000 | Invitrogen | #PA1-186 | WB |  |
|  | 1:200 |  |  | IF |  |
|  | 1:300 |  |  | IHC-P |  |
| **LOX-1** | 1:1000 | Invitrogen | #PA5-102452 | WB |  |
|  | 1:100 |  |  | IF/ IHC-P |  |
| **Anti-Actin, α-Smooth Muscle - Cy3™ antibody, Mouse monoclonal** | 1:1000 | Sigma | C6198 | IF |  |
| **Mouse/Rat CD31/PECAM-1 Antibody** | 1:200 | R&D System | AF3628 | IF |  |
| **DAPI** | 1:1000 | Thermo Scientific | 62248 | IF |  |
| **β-Actin** | 1:5000 | Sigma Aldrich | A5441 | WB |  |
| **555 Goat anti-rabbit** | 1:500 | Invitrogen | A32732 | IF |  |
| **Horse Anti-Rabbit IgG (H+L), Biotinylated** | 1:200 | Vector Laboratories | BA-1100-1.5 | IHC-P |  |
| **Goat Anti-Rabbit IgG (H+L), Horseradish Peroxidase (HRP) Conjugate, affinity purified** | 1:3000 | Invitrogen | A16096 | WB |  |
|  | 1:200 |  |  | IHC |  |
| **Horse Anti-Mouse IgG (H+L), Biotinylated** | 1:200 | Vector Laboratories | BA-2001 | IHC-P |  |
| **ECL^TM^ Anti-Mouse IgG, Horseradish Peroxidase linked whole antibody (from sheep)** | 1:5000 | Sigma Aldrich | NA931V | WB |  |
| **Alexa Fluor^TM^ 594 donkey anti-rabbit IgG (H+L)** | 1:200 | Thermo Scientific | A21207 | IF |  |
| **Alexa Fluor^TM^ 488 donkey anti-rabbit IgG (H+L)** | 1:200 | Thermo Scientific | A21206 | IF |  |
|  |  |  |  |  |  |
